# MiR-135-5p-p62 Axis Regulates Autophagic Flux, Tumorigenic Potential, and Cellular Interactions Mediated by Extracellular Vesicles During Allergic Inflammation

**DOI:** 10.3389/fimmu.2019.00738

**Published:** 2019-04-05

**Authors:** Misun Kim, Yeongseo Park, Yoojung Kwon, Youngmi Kim, Jaehwan Byun, Myeong Seon Jeong, Han-Ul Kim, Hyun Suk Jung, Ji Young Mun, Dooil Jeoung

**Affiliations:** ^1^Department of Biochemistry, Kangwon National University, Chuncheon, South Korea; ^2^Chuncheon Center, Korean Basic Science Institute, Chuncheon, South Korea; ^3^Department of Structure and Function of Neural Network, Korea Brain Research Institute, Daegu, South Korea

**Keywords:** P62, miR-135, extracellular vesicles, cellular interactions, allergic inflammation

## Abstract

The objective of this study was to investigate the relationship between autophagy and allergic inflammation. *In vitro* allergic inflammation was accompanied by an increased autophagic flux in rat basophilic leukemia (RBL2H3) cells. 3-MA, an inhibitor of autophagic processes, negatively regulated allergic inflammation both *in vitro* and *in vivo*. The role of p62, a selective receptor of autophagy, in allergic inflammation was investigated. P62, increased by antigen stimulation, mediated *in vitro* allergic inflammation, passive cutaneous anaphylaxis (PCA), and passive systemic anaphylaxis (PSA). P62 mediated cellular interactions during allergic inflammation. It also mediated tumorigenic and metastatic potential of cancer cells enhanced by PSA. TargetScan analysis predicted that miR-135-5p was a negative regulator of p62. Luciferase activity assay showed that miR-135-5p directly regulated p62. MiR-135-5p mimic negatively regulated features of allergic inflammation and inhibited tumorigenic and metastatic potential of cancer cells enhanced by PSA. MiR-135-5p mimic also inhibited cellular interactions during allergic inflammation. Extracellular vesicles mediated allergic inflammation both *in vitro* and *in vivo*. Extracellular vesicles were also necessary for cellular interactions during allergic inflammation. Transmission electron microscopy showed p62 within extracellular vesicles of antigen-stimulated rat basophilic leukemia cells (RBL2H3). Extracellular vesicles isolated from antigen-stimulated RBL2H3 cells induced activation of macrophages and enhanced invasion and migration potential of B16F1 mouse melanoma cells in a p62-dependent manner. Extracellular vesicles isolated from PSA-activated BALB/C mouse enhanced invasion and migration potential of B16F1 cells, and induced features of allergic inflammation in RBL2H3 cells. Thus, miR-135-5p-p62 axis might serve as a target for developing anti-allergy drugs.

## Introduction

Impaired autophagy in myeloid cells has a causal role in eosinophilic inflammation and chronic rhino sinusitis (1). Dysregulation of autophagy and inflammasome activity contributes to the development of auto-inflammatory diseases (2). Autophagy plays a crucial role in degranulation of mast cells (3, 4). Histamine H3 receptor (H3R) blockade can inhibit mammalian target of rapamycin (mTOR) phosphorylation and reinforce autophagy (5). B cell autophagy aggravates experimental asthma through multiple mechanisms (6). Antibody-enhanced Dengue viruses (DENV) infection of KU812 cells (pre-basophil-like cell line) and immature human mast cell line (HMC-1) shows increases of autophagosome vesicles, light chain 3 (LC3) punctation, and LC3-II accumulation (7). MTOR, an inhibitor of autophagy, mediates metabolic adaptation of antigen presenting cells (APCs) in distinct tissues, thus influencing immunological characters of allergic inflammation (8). Inhibition of PI3K/Akt activity and subsequent blockade of mTOR-hypoxia inducible factor (HIF)-1α-vascular endothelial growth factor (VEGF) module can attenuate typical asthmatic attack in a murine model (9). Transglutaminase II (TGaseII) mediates passive cutaneous anaphylaxis and atopic dermatitis (10). TGase II through interaction with NF-kappaB can induce histone deacetylase-3 (HDAC3) by direct binding to promoter sequences (10). HDAC3 can interact with FcεRIβ and mediate allergic inflammation by increasing expression of monocyte chemo attractant protein 1 (MCP1) (11). Down-regulation of HDAC3 abrogates the ability of HDAC inhibitor valproic acid (VPA) to modulate AKT phosphorylation, suppress tumor cell growth, and induce autophagy (12). Thus, autophagy might play a role in allergic inflammation.

Scaffolding adaptor protein P62/SQSTM1 is an autophagy receptor that acts as a link between ubiquitination and autophagy machineries. Upon binding to its ligand, p62 acts as a modulator of macroautophagy and induces autophagosome biogenesis (13). Stimulation of TLR2/6 or TLR4 in primary human keratinocytes can activate autophagy pathways and increase p62 expression through induction of NADPH oxidases 2 and 4 and generation of reactive oxygen species (14). P62 acts downstream of TCR activation. It is important for Th2 polarization and asthma. P62 also plays a significant role in the control of sustained activation of NF-kappaB and late synthesis of GATA3 and IL-4 by participating in the activation of the IKK complex (15). Overexpression of p62 increases expression levels of pro-inflammatory cytokines, such as TNFα, CXCL10, and CCL2 (16). P62 can stabilize COX-2 protein through its ubiquitin-associated domain. P62 can also regulate prostaglandin E_2_ production *in vitro* (17). It is known that miR-26a/-26b-COX-2 axis regulates allergic inflammation (18). These reports suggest a role of p62 in allergic inflammation.

Asthma shows enhanced secretion of extracellular vesicles by epithelial cells, not by macrophages, under the influence of IL-13 (19). Alveolar macrophages secrete SOCS1 and SOCS3 in extracellular vesicles and microparticles, respectively, for uptake by alveolar epithelial cells and subsequent inhibition of STAT activation (20). MiR-122-SOCS1 axis regulates allergic inflammation (21). Increased release of extracellular vesicles can induce autophagy (22). BALF extracellular vesicles from asthmatics might contribute to subclinical inflammation by increasing generation of cytokine and LTC (4) in airway epithelium (23). GW4869, an inhibitor of extracellular vesicles formation, can decrease Th2 cytokines and eosinophil counts in BALFs and reduce eosinophil accumulation in airway walls and mucosa (24). These reports suggest a role of extracellular vesicles in allergic inflammation.

In this study, we present a novel role of miR-135-5p-p62 axis in regulating allergic inflammation in conjunction with autophagic flux, cellular interactions, and allergic inflammation-promoted enhanced tumorigenic and metastatic potential of cancer cells. We showed the presence of p62 within extracellular vesicles and the role of p62 in cellular interactions mediated by extracellular vesicles during allergic inflammation. Thus, miR-135-5p-p62 axis can be employed to develop anti-allergy therapeutics.

## Materials and Methods

### Materials

Oligonucleotides used in this study were commercially synthesized by the Bioneer Co. (Daejeon Korea). DNP-HSA (2,4-dinitrophenyl-human serum albumin), TNP-BSA (trinitrophenyl-bovine serum albumin), DNP-specific IgE antibody, and TNP-specific IgE antibody were purchased from Sigma. Chemicals used in this study were purchased from Sigma. All other antibodies were purchased from Cell Signaling Co. (Beverly, MA). Anti-mouse and anti-rabbit IgG-horseradish peroxidase-conjugated antibody was purchased from Pierce. Lipofectamine and PlusTM reagent for transfection were purchased from Invitrogen.

### Cell Culture

Rat basophilic leukemia (RBL2H3) cells, B16F1 cells, and B16F10 cells were obtained from the Korea Cell Line Bank (Seoul, Korea). Cells were grown in Dulbecco's modified Eagle's medium containing heat-inactivated fetal bovine serum, 2 mM L-glutamine, 100 units/ml penicillin, and 100 μg/ml streptomycin (Invitrogen). Cultures were maintained in 5% CO_2_ at 37°C. Lung mast cells and lung macrophages were isolated according to standard procedures (25).

### Mice

Five-weeks-old female BALB/C mice were purchased from Nara Biotech (Seoul, Korea). All animal experiments were approved by the Institutional Animal Care and Use Committee (IACUC) of Kangwon National University (KIACUC-160329-2) and conducted in accordance with the ethical committee guidelines for the care and use of laboratory animals. To measure tumorigenic potential, mouse melanoma B16F1 cells (1 × 10^6^ cells in 100 μl of PBS), after induction of passive systemic anaphylaxis, were injected subcutaneously into the right flank of each mouse (*n* = 5).

### β-Hexosaminidase Activity Assays

The β-hexosaminidase activity assay was performed according to standard procedures (26).

### Immunoblot and Immunoprecipitation

Immunoblot and immunoprecipitation were performed according to the standard procedures (25).

### The Levels of PGE2 and Histamine Release

The levels of PGE2 and the amount of histamine released were measured according to the manufacturer's instruction using commercially available ELISA kit (Abcam, UK). Reaction product was measured colorimetrically with a microplate reader.

### Chemo Invasion and Migration Assays

The invasive potential was determined by using a transwell chamber system with 8-μm pore polycarbonate filter inserts (CoSTAR, Acton, MA). The lower and upper sides of the filter were coated with gelatin and matrigel, respectively. For determination of migration potential, the lower sides of the filters were coated with gelatin. Trypsinized cells (5 × 10^3^) in the serum-free RPMI 1640 medium containing 0.1% bovine serum albumin were added to each upper chamber of the transwell. RPMI 1640 medium supplemented with 10% fetal bovine serum was placed in the lower chamber and cells were incubated at 37°C for 16 h. The cells were fixed with methanol and the invaded cells were stained and counted.

### Immunofluorescence Staining

Cells were seeded onto glass coverslips in 24-well plates and were fixed with 4% paraformaldehyde (v/v) for 10 min and then permeabilized with 0.4% Triton X-100 for 10 min. Cells were incubated with primary antibody specific to LC3 (1:100; Santa Cruz Biotechnology), P62 (1:100; Santa Cruz Biotechnology), CD163 (1:100; Ab Cam) or iNOS (1:100; Santa Cruz Biotechnology) for 2 h. Anti-rabbit Alexa Fluor 488 (for detection of LC3 and iNOS) or anti-goat Alexa Fluor 546 (for detection of P62 and CD163) secondary antibody (Molecular Probes) was added to cells and incubated for 1 h. Fluorescence images were acquired using a confocal laser scanning microscope and software (Fluoview version 2.0) with a X 60 objective (Olympus FV300, Tokyo, Japan).

### Matrigel Plug Assays

Seven weeks-old BALB/C mice (Nara Biotech) were injected subcutaneously with 0.1 ml of matrigel containing culture medium and 10 units of heparin (Sigma). After 8 days, the skin of the mouse was easily pulled back to expose the matrigel plug, which remained intact. Hemoglobin (Hb) content in the matrigel plugs was measured using the Drabkin reagent (Sigma, USA) for quantification of blood vessel formation.

### Transfection

Transfections were performed according to the manufacturer's instructions. Lipofectamine and Plus reagents (Invitrogen) were used. For miR-135-5p knockdown, cells were transfected with 10 nM oligonucleotide (inhibitor) with Lipofectamine 2000 (Invitrogen), according to the manufacturer's protocol. The sequences used were 5′-UUCACAUAGGAAUAAAAAGCCAUA-3′ (miR-135-5p inhibitor) and 5′-TAACACGTCTATACGCCCA-3′ (control inhibitor).

### miRNA Target Analysis

Genes that contain the miRNA-binding site(s) in the UTR were obtained using the TargetScan program (http://www.targetscan.org/, http://pictar.mdc-berlin.de/, http://www.microrna.org/microrna/home.do).

### RNA Extraction and Quantitative Real Time PCR (QRT-PCR)

Total miRNA was isolated using the *mir*VanamiRNA isolation kit (Ambion). MiRNA was extended by a poly (A) tailing reaction using the A-Plus poly (A) polymerase tailing kit (Cell Script). cDNA was synthesized from miRNA with poly(A) tail using a poly(T) adaptor primer and qScriptTM reverse transcriptase (Quanta Biogenesis). Expression level of miR-135-5p or p62 was quantified with SYBR Green quantitative real-time-PCR kit (Ambion) using miRNA-specific forward primer and a universal poly (T) adaptor reverse primer. Expression level of miR-135-5p was defined based on the threshold (*Ct*), and relative expression levels were calculated as 2^−(Ct*ofmiR*−135−5*p*)−(*CtofU*6)^ after normalization with reference to expression of U6 small nuclear RNA. For quantitative real-time PCR, SYBR PCR Master Mix (Applied Biosystems) was used in a CFX96 Real Time System thermocycler (Bio-Rad).

### Constructs

To generate the pGL3–3′-UTR-P62 construct, a (136)-bp human p62 gene segment encompassing 3′-UTR was PCR-amplified and subcloned into the (XbaI) site of pGL3 luciferase plasmid. The mutant pGL3–3′-UTR-CAGE construct was made with the QuikChange site-directed mutagenesis kit (Stratagene). Luciferase activity assay was performed according to the instruction manual (Promega).

### Passive Cutaneous Anaphylaxis

BALB/C mice were sensitized with an intradermal injection of IgE (0.5 μg/kg). Twenty four hours later, mice were challenged with an intravenous injection of DNP-HSA (250 μg/kg) and 2% (v/v) Evans blue solution. One hour after injection with evans blue solution after DNP-HSA challenge, the mice were euthanized, and the 2% (v/v) Evans blue dye was extracted from each dissected ear in 700 μl of acetone/water (7:3) overnight. The absorbance of Evans blue in the extracts was measured with a spectrophotometer at 620 nm. To determine the effect of p62 on the PCA, BALB/C mice were given an intradermal injection of DNP-IgE (0.5 μg/kg) and intravenous injection of p62 siRNA (100 nM). The next day, BALB/C mice were given an intravenous injection of PBS or DNP-HSA (250 μg/kg) along with 2% (v/v) Evans blue solution for determining the extent of vascular permeability accompanied by PCA.

### Effect of Passive Systemic Anaphylaxis on Tumorigenic Potential

BALB/C mice were sensitized by intravenous injection of IgE (0.5 μg/kg). The next day, the sensitized mice were intravenously injected with DNP-HSA (250 μg/kg). Two days after injection of DNP-HSA, B16F1 mouse melanoma cells (1 × 10^6^ cells) were injected into the flanks of each BALB/C mouse. To determine the effect of p62 on the enhanced tumorigenic potential by PSA, BALB/C mice were given an intravenous injection with p62 siRNA (100 nM) on the indicated days.

### Effect of Passive Systemic Anaphylaxis on Metastatic Potential

Passive systemic anaphylaxis was induced as described. Three days after the injection of IgE, BALB/C mice were given an intravenous injection of B16F1 melanoma cells (2 × 10^5^). To determine the effect of p62 on the enhanced metastatic potential of cancer cells by PSA, BALB/C mice were given an intravenous injection with p62 siRNA (100 nM) on days 5, 7, 10 and 12. On day 14, lung tumor tissues were harvested.

### Monitoring of Rectal Temperature

Changes in core body temperature associated with systemic anaphylaxis were monitored by measuring changes in rectal temperatures using a rectal probe coupled to a digital thermometer.

### Immunohistochemical Staining

Immunohistochemical staining was performed using avidin-biotin detection method (Vectastain ABC kit, Vector Laboratories Inc., Burlingame, CA).

### Electron Microscopic Observation of Autophagosomes

The IgE-sensitized RBL2H3 cells stimulated without or with DNP-HSA (100 ng/ml) for 2 h were fixed with 2.5% glutaraldehyde in 0.1 M cacodylate solution (pH 7.0) for 1 h, and then followed with 2% osmium tetroxide for 2 h at 4°C. Then, the cells were dehydrated with a graded acetone series, and embedded into Spurr medium (Electron Microscopy System). The samples were sectioned (60 nm) with an ultra-microtome (RMC MTXL, Arizona, USA), and double-stained with 2% uranyl acetate for 20 min and lead citrate for 10 min. The sections were then viewed under a Tecnai G2 (FEI, USA) TEM at 200 kV.

### Isolation and Characterization of Extracellular Vesicles

Cells were cultured under serum-free medium (Invitrogen, Carlsbad, CA). The culture medium was harvested after 48 h of incubation, and the extracellular vesicles fraction was purified using Exoquick-TC reagent (System Biosciences, Mountain View, CA) according to the manufacturer's instructions. Extracellular vesicles were observed under a Tecnai T10 transmission electron microscope (FEI, USA).

### Labeling and Internalization of Extracellular Vesicles

Extracellular vesicles from antigen-stimulated RBL2H3 cells were isolated and were labeled using PKH67 Fluorescent Cell Linker kits (Sigma-Aldrich, St. Louis, MO). To examine the uptake of extracellular vesicles, unstimulated RBL2H3 cells were plated out onto coverslip (2 × 10^4^ cells). After 24 h, coverslips were washed three times in PBS, and each medium containing PKH67-labeled extracellular vesicles or PKH67-unlabeled extracellular vesicles were added into each well for 24 h. After incubation, the coverslips were washed three times in PBS, and 4% paraformaldehyde solution then added to the slides for 15 min. The coverslips were washed three times in PBS. Cells were visualized under a confocal laser scanning microscope LX70 FV300 05-LPG-193 (Olympus).

### The Presence of P62 in the Extracellular Vesicles of Antigen-Stimulated RBL2H3 Cells

Extracellular vesicles extracted from antigen-stimulated RBL2H3 cells (REF, KIT model) were subjected to centrifugation at 60,000 g for 30 min to precipitate extracellular vesicles. Collected extracellular vesicles were fixed with 0.1% glutaraldehyde and 2% paraformaldehyde in phosphate buffer (pH 7.4) for 1 h at 4°C and then post-fixed in 2% osmium tetroxide for 30 min at 4°C. They were dehydrated with a graded series of ethanol followed by treatment with graded propylene oxide series, and embedded into epoxy resin (PELCO, USA). Ultrathin sections (~80 nm) were obtained with Ultracut UCT (Leica, Germany), mounted on copper grids, and stained with 1% uranyl acetate and lead citrate (10 min) for the subsequent observations. For immune-gold labeling electron microscopy, ultrathin sections on the grids were treated with 0.02 M glycine for 10 min for quenching the reaction of free aldehyde group. Sections were then washed in deionized water, floated for 1 h in PBS containing 1% BSA, and incubated directly in the primary rabbit or/and mouse antibodies (Anti-P62 or/and Anti-CD63 antibodies) at 1:20 dilutions for overnight at 4°C. The grid were washed five time with 0.1% BSA in PBS, incubated in secondary antibodies, anti- Rabbit IgG conjugated to 10 nm and anti-mouse IgG conjugated to 25 nm (AURION, Holland) diluted 1:20 in 0.1% BSA-PBS. The sample grids were stained with uranyl acetate and lead citrate. The sectioned and immune-gold labeled grids were examined using a Tecnai T10 transmission electron microscope (FEI, USA) operated at 100 kV and JEOL-2100F transmission electron microscope (JEOL, USA) operated at 200 KV.

### Statistical Analysis

Data were analyzed and graphed using GraphPad Prism statistics program (GraphPad Prism software). Results are presented as means ± S.E. Statistical analysis was performed using one way *t*-tests with differences between means considered significant when *p* < 0.05.

## Results

### P62 Mediates Allergic Inflammation by Regulating Autophagic Flux

**Figure 1:**
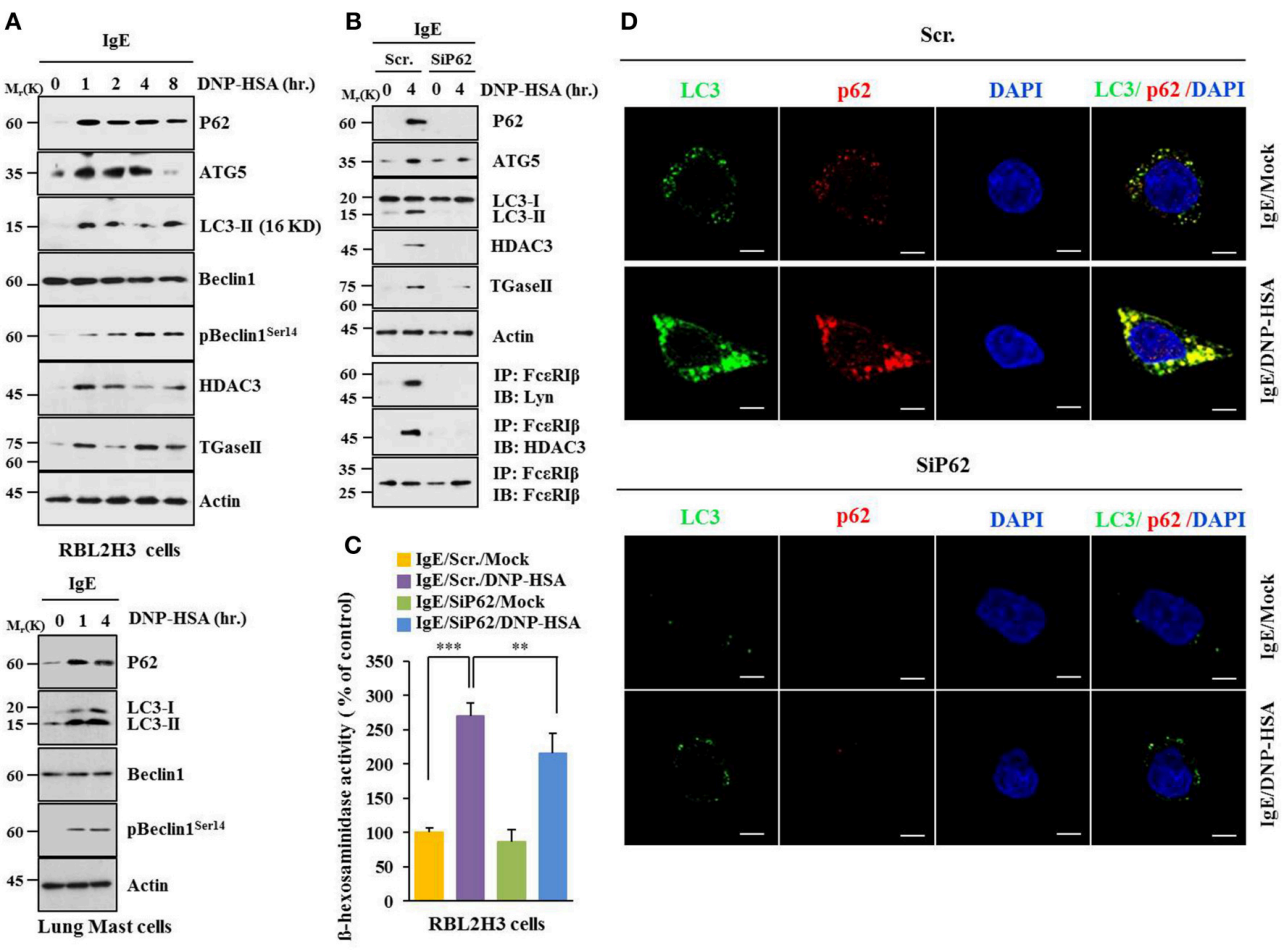
P62 is necessary for allergic inflammation and regulation of autophagic flux**. (A)** IgE (DNP-specific)-sensitized RBL2H3 cells were treated without or with DNP-HSA (100 ng/ml) for various time intervals followed by immunoblot (upper panel). IgE-sensitized lung mast cells were treated without or with DNP-HSA for various time intervals followed by immunoblot (lower panel). Each blot is a representative of three independent experiments. **(B)** RBL2H3 cells were transfected with indicated siRNA (each at 10 nM). The next day, cells were sensitized with IgE for 24 h followed by stimulation without or with DNP-HSA. Scr. denotes scrambled siRNA. Each blot is a representative of three independent experiments. **(C)** Same as **(B)** except that ß-hexosaminidase activity was performed. ^**^p < 0.005; ^***^p < 0.0005. Each value represents average of three independent experiments. **(D)** Immunofluorescence staining shows co-localization of p62 with LC3 in RBL2H3 cells. Scale bars represent 10 μm.

Based on a close relationship between allergic inflammation and autophagy (3, 4, 26) and the role of p62, a selective adaptor in autophagic processes (13), effect of p62 on allergic inflammation was examined. Antigen DNP-HSA increased autophagic flux, such as ATG5, LC3-II, pBeclin1^Ser14^, and p62, along with HDAC3 and TGaseII in RBL2H3 cells (Figure 1A). Roles of HDAC3 and TGaseII in allergic inflammation have been previously reported (11, 25, 26). Expression levels of LC3-II, p62, and pBeclin1^Ser14^ were increased in antigen-stimulated lung mast cells (Figure 1A, lower panel). Down-regulation of p62 prevented antigen from increasing autophagic flux, HDAC3, and TGaseII. It prevented antigen from inducing interactions of FcεRIß with Lyn and HDAC3 (Figure 1B). It also prevented antigen from increasing ß-hexosaminidase activity (Figure 1C). Down-regulation of p62 only blocked the increase of p62 expression upon allergen stimulation, but not decreased it below the level detected in non-stimulated RBL2H3 cells (Figure 1B). Increased LC3 puncta expression (Figure 1D) and co-localization of p62 with LC3 were seen in antigen-stimulated RBL2H3 cells (Figure 1D). Antigen-stimulated RBL2H3 cells showed increased number of autolysosomes compared to un-stimulated RBL2H3 cells (Figure S1). 3-MA, an inhibitor of autophagic processes, prevented antigen from increasing levels of autophagic flux and hallmarks of allergic inflammation, prevented antigen from inducing interactions of FcεRIß with Lyn and HDAC3 (Figure S2A), prevented antigen from increasing ß-hexosaminidase activity (Figure S2B), inhibited passive cutaneous anaphylaxis (PCA) (Figure S2C), prevented antigen from increasing ß-hexosaminidase activity (Figure S2D), and prevented antigen from increasing autophagic flux and hallmarks of allergic inflammation in a mouse model of PCA (Figure S2E). Thus, allergic inflammation is mediated by p62 which regulates autophagic flux. Effect of p62 on allergic inflammation in conjunction with autophagic flux has not been reported previously.

### P62 Mediates Anaphylaxis

**Figure 2:**
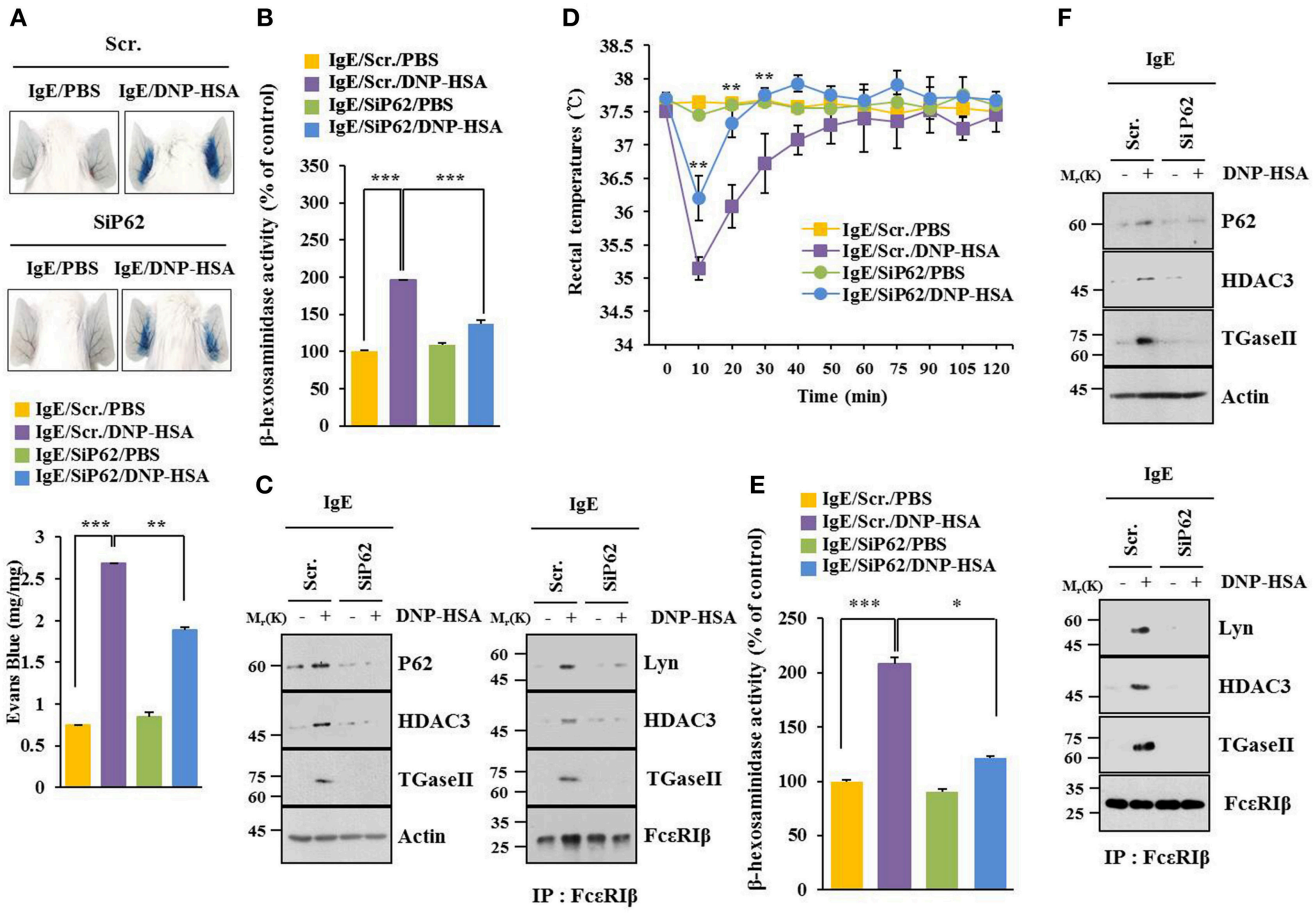
P62 mediates anaphylaxis. **(A)** BALB/C mice were given an intradermal injection of IgE (0.5 μg/kg) and an intravenous injection of indicated siRNA (each 100 nM). The next day, BALB/C mice were given an intravenous injection of PBS or DNP-HSA (250 μg/kg) along with 2% (v/v) Evans blue solution. Representative images of each BALB/C mouse of each experimental group are shown. Each experimental group consisted of four BALB/C mice. **(B,C)** Ear tissue lysate from BALB/C mouse of each experimental group was subjected to β-hexosaminidase activity assay, immunoblot, and immunoprecipitation. **(D)** BALB/C mice were given an intravenous injection with indicated siRNA. The next day, BALB/C mice were given an intravenous injection with IgE. The following day, BALB/C mice were given an intravenous injection with DNP-HSA and rectal temperatures were measured. Each experimental group consisted of four BALB/C mice. Means ± S.E. of three independent experiments are depicted. Comparison was made between PSA-activated mice and mice injected with SiP62. **(E,F)** Tissue lysates were subjected to β-hexosaminidase activity assay, immunoblot, and immunoprecipitation. ^*^p < 0.05; ^**^p < 0.005; ^***^p < 0.005.

BALB/C mouse model of passive cutaneous anaphylaxis (PCA) was employed to investigate the role of p62 in allergic inflammation. PCA increased vascular permeability (Figure 2A) and β-hexosaminidase activity (Figure 2B) in a p62-dependent manner. P62 was necessary for increased expression levels of HDAC3 and TGase II. It was also necessary for interactions of FcεRIβ with HDAC3, Lyn, and TGaseII in a mouse model of PCA (Figure 2C). Passive systemic anaphylaxis (PSA) decreased rectal temperatures of BALB/C mice (Figure 2D), but increased β-hexosaminidase activity (Figure 2E) in a p62-dependent manner. Down-regulation of p62 prevented antigen from increasing expression levels of HDAC3 and TGaseII. It also prevented antigen from inducing interactions of FcεRIβ with HDAC3, TGase II, and Lyn (Figure 2F). Thus, p62 can mediate anaphylaxis *in vivo*.

### P62 Mediates Tumorigenic Potential of B16F1 Cells Enhanced by Passive Systemic Anaphylaxis

**Figure 3:**
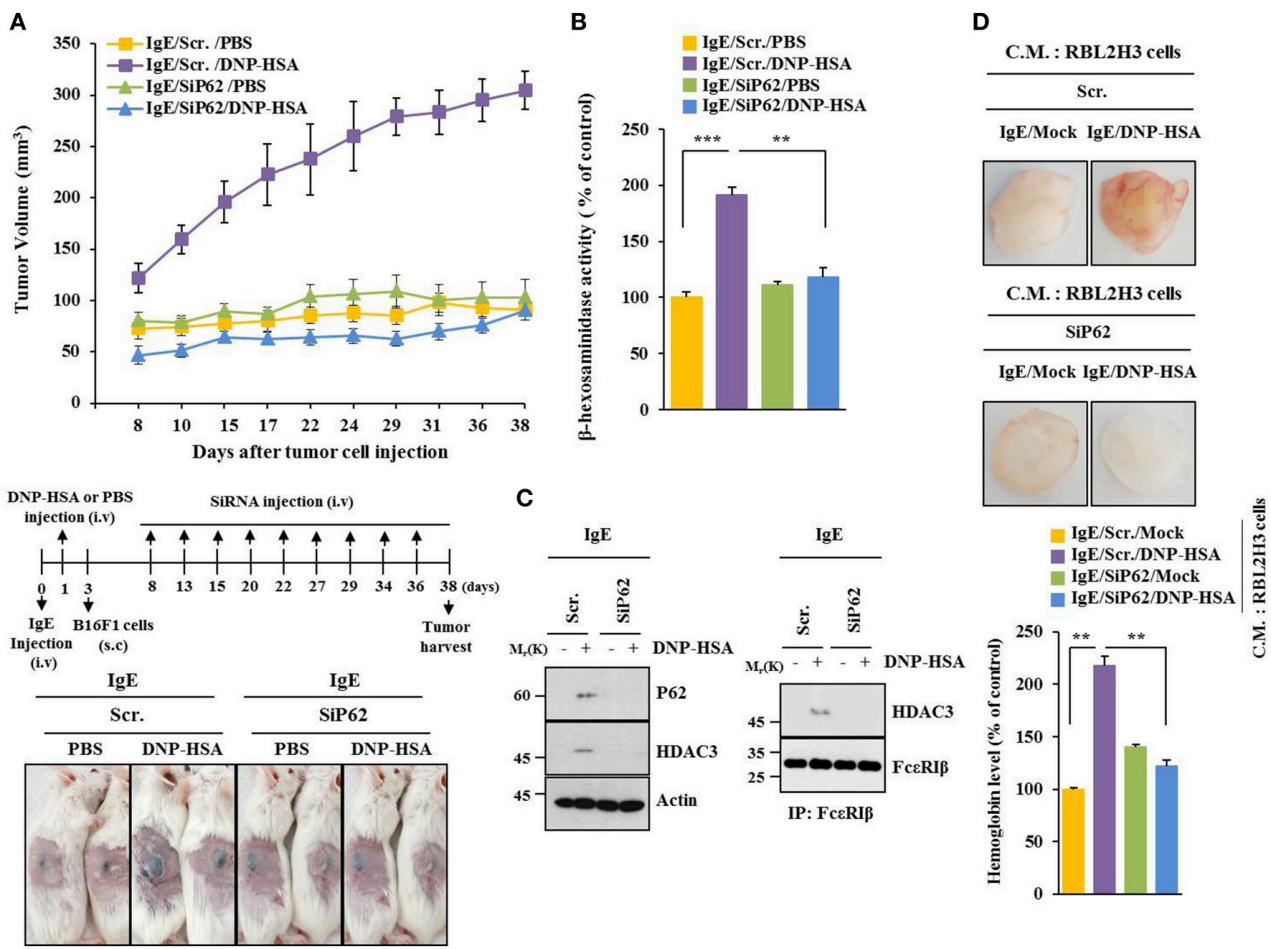
P62 mediates tumorigenic potential of B16F1 cells enhanced by passive systemic anaphylaxis. **(A)** Passive systemic anaphylaxis (PSA) was induced as described. Each mouse received injection of B16F1 melanoma cells (2 × 10^5^) on day 3. After tumor reached a certain size, BALB/C mice were given an intravenous injection of indicated siRNA. Each experimental group consisted of four BALB/C mice. **(B,C)** Tumor tissue lysate from each experimental group was subjected to β-hexosaminidase activity assays, immunoblot, and immunoprecipitation. **(D)** Culture medium of antigen-stimulated RBL2H3 cells transfected with each siRNA for 48 h was subjected to matrigel plug assays. C.M. denotes culture medium. ^**^p < 0.005; ^***^p < 0.005.

PSA enhanced tumorigenic potential of B16F1 cells (Figure 3A) and increased β-hexosaminidase activity in a p62-depedent manner (Figure 3B). PSA induced interaction between FcεRIβ and HDAC3 in a p62-dependent manner (Figure 3C). Enhanced tumorigenic potential by allergic inflammation is known to be due to enhanced angiogenic potential during allergic inflammation (21). Culture medium of antigen-stimulated RBL2H3 cells showed angiogenic potential in a p62-dependent manner based on matrigel plug assays (Figure 3D). Thus, p62 can mediate allergic inflammation-promoted enhancement in tumorigenic potential of cancer cells.

### P62 Mediates Metastatic Potential of B16F1 Cells Enhanced by Passive Systemic Anaphylaxis

**Figure 4:**
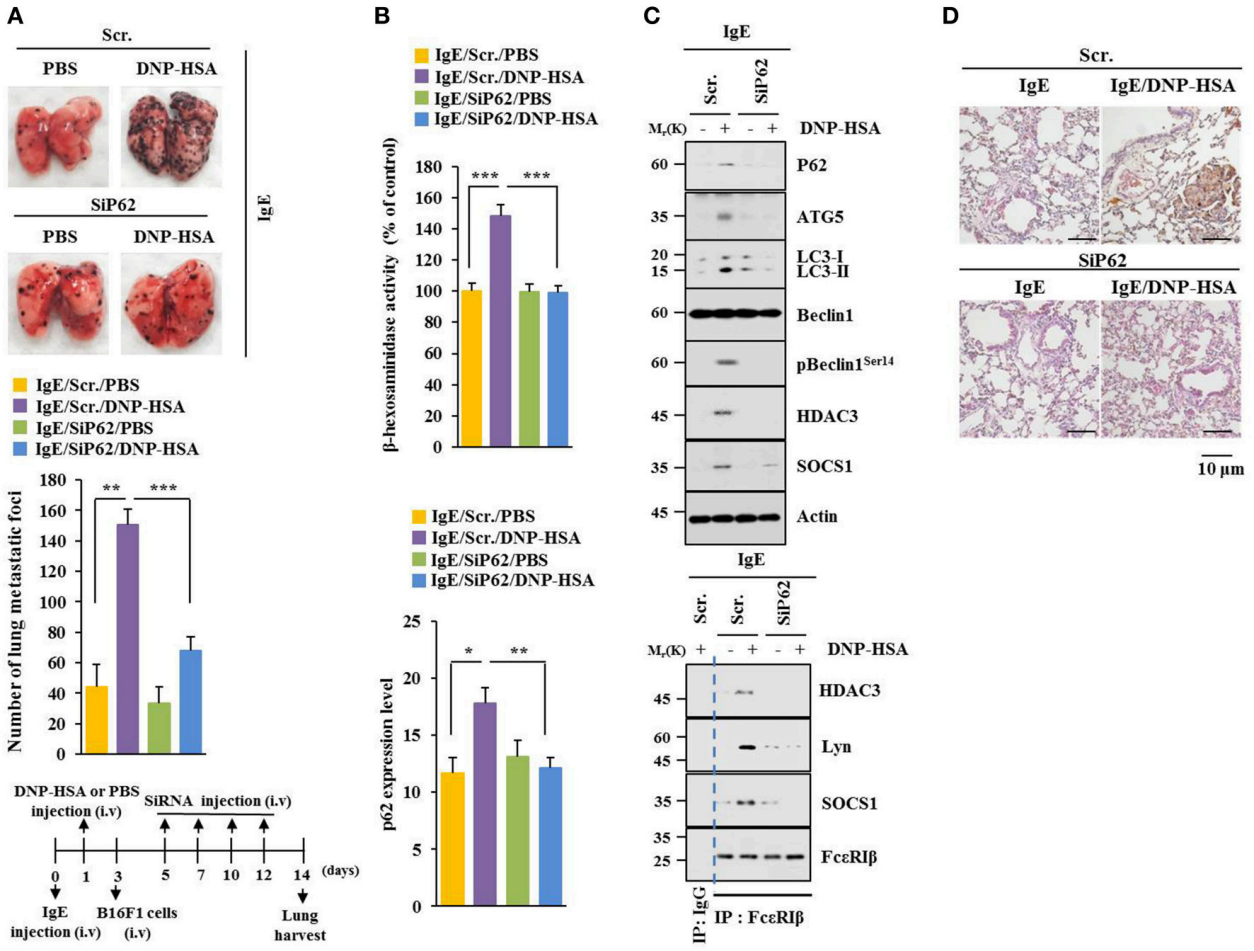
P62 mediates metastatic potential of B16F1 cells enhanced by passive systemic anaphylaxis. **(A)** Each mouse received an injection of B16F1 melanoma cells (2 × 10^5^) on day 3. The extent of lung metastasis was determined. H&E staining was also performed. Each experimental group consisted of four BALB/C mice. **(B,C)** Lung tumor tissue lysates were subjected to β-hexosaminidase activity assays, qRT-PCR analysis, immunoblot, and immunoprecipitation. **(D)** Immunohistochemical staining of lung tumor tissue employing p62 antibody was performed. ^*^p < 0.05; ^**^p < 0.005; ^***^p < 0.005.

P62 can promote tumor cell growth and metastasis in a Twist1-dependent manner (27). PSA enhanced metastatic potential of B16F1 cells (Figure 4A) and increased β-hexosaminidase activity in a p62-dependent manner (Figure 4B). Down-regulation of p62 prevented PSA from increasing levels of autophagic flux and hallmarks of allergic inflammation. It also prevented PSA from inducing interactions of FcεRIβ with HDAC3, Lyn, and SOCS1 (Figure 4C). Immunohistochemical staining showed increased expression level of p62 by PSA (Figure 4D). Thus, p62 can mediate enhanced metastatic potential of cancer cells by allergic inflammation.

### P62 Mediates Cellular Interactions During Allergic Inflammation

**Figure 5:**
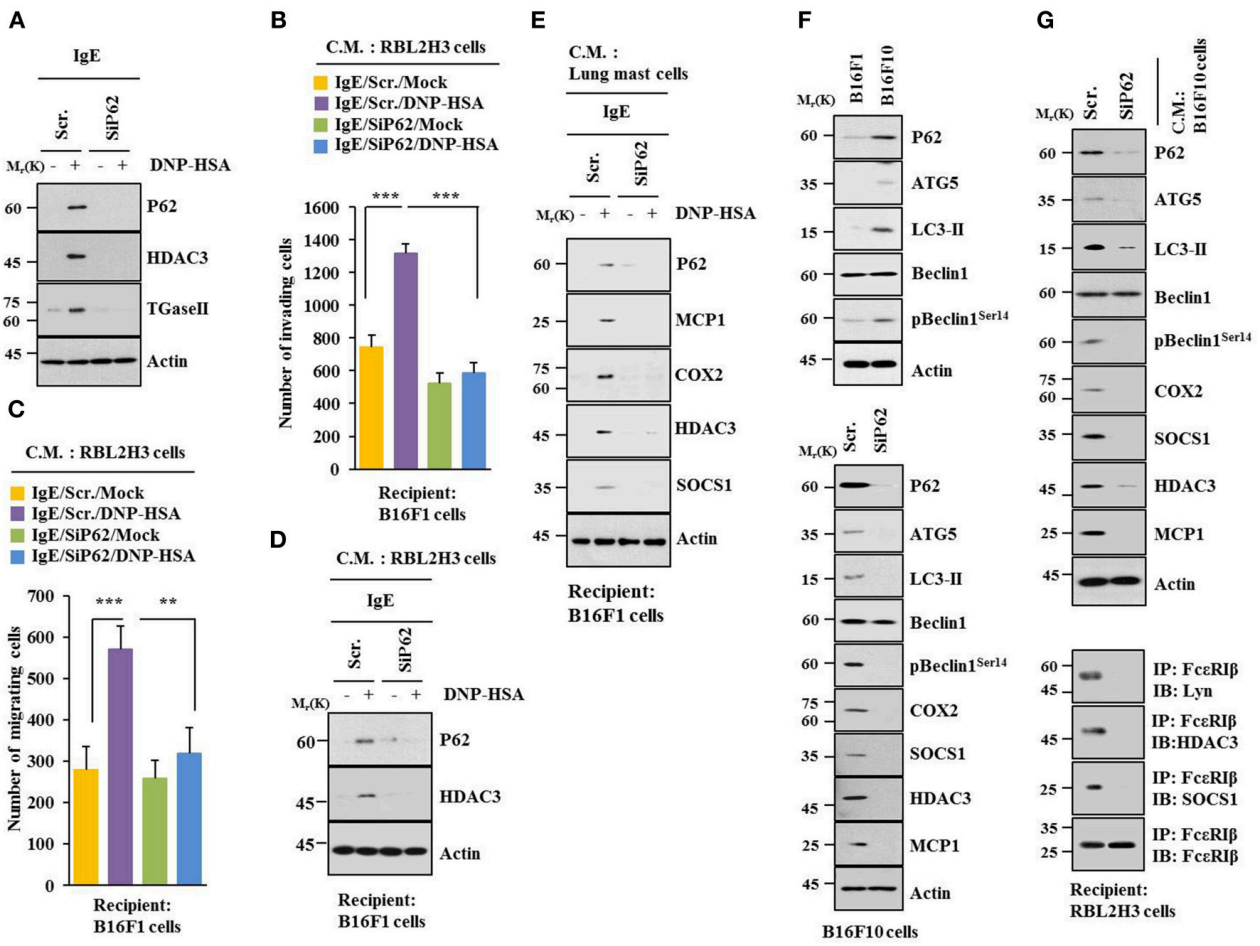
P62 mediates cellular interactions during allergic inflammation. **(A)** Immunoblot was performed. **(B–D)** One hour after stimulation with DNP-HSA, culture medium was added to B16F1 cells and incubated for 8 h followed by migration, invasion assays, and immunoblot. C.M. denotes culture medium. **(E)** Same as **(D)** except that culture medium of lung mast cells was employed. **(F)** Cell lysates from indicated cells were subjected to immunoblot (upper panel). At 48 h after transfection with indicated siRNA, immunoblot was performed (lower panel). **(G)** At 48 h after transfection with indicated siRNA, culture medium of B16F10 cells was added to RBL2H3 cells and incubated for 8 h followed by immunoblot and immunoprecipitation. ^**^p < 0.005; ^***^p < 0.005.

Allergic inflammation-enhanced tumorigenic and metastatic potentials of cancer cells are known to be due to interactions between cancer cells and immune cells, such as mast cells and macrophages (11, 25, 26). Antigen increased expression levels of HDAC3 and TGaseII in RBL2H3 cells in a p62-dependent manner (Figure 5A). When culture medium of antigen-stimulated RBL2H3 cells was added to B16F1 cells, it increased invasion (Figure 5B), migration potential (Figure 5C), and expression level of HDAC3 (Figure 5D) in a p62-dependent manner. When culture medium of antigen-stimulated lung mast cells was added to B16F1 cells, it increased expression levels of hallmarks of allergic inflammations, such as MCP1, COX2, HDAC3, and SOCS1 in a p62-dependent manner (Figure 5E). B16F10 cells showed higher level of autophagic flux than B16F1 cells (Figure 5F). Down-regulation of p62 decreased autophagic flux and hallmarks of allergic inflammation in B16F10 cells (Figure 5F). When culture medium of B16F10 cells was added to RBL2H3 cells, it increased hallmarks of allergic inflammation and autophagic flux. It also induced interactions of FcεRIß with HDAC3, Lyn, and SOCS1 in a p62-dependent manner (Figure 5G). When culture medium of B16F10 cells was added to lung macrophages, it increased hallmarks of allergic inflammation and autophagic flux in a p62-dependent manner (Figure S3A). When culture medium of B16F10 cells (Figure S3B) or RBL2H3 cells (Figure S3D) was added to lung macrophages, it increased expression level of CD163, but decreased expression level of iNOS in a p62-dependent manner. When culture medium of RBL2H3 cells was added to lung macrophages, it increased hallmarks of allergic inflammation, autophagic flux, and CD163, but decreased expression level of iNOS in a p62-dependent manner (Figure S3C). Thus, p62 can mediate cellular interactions during allergic inflammation.

### MiR-135-5p Directly Targets p62

**Figure 6:**
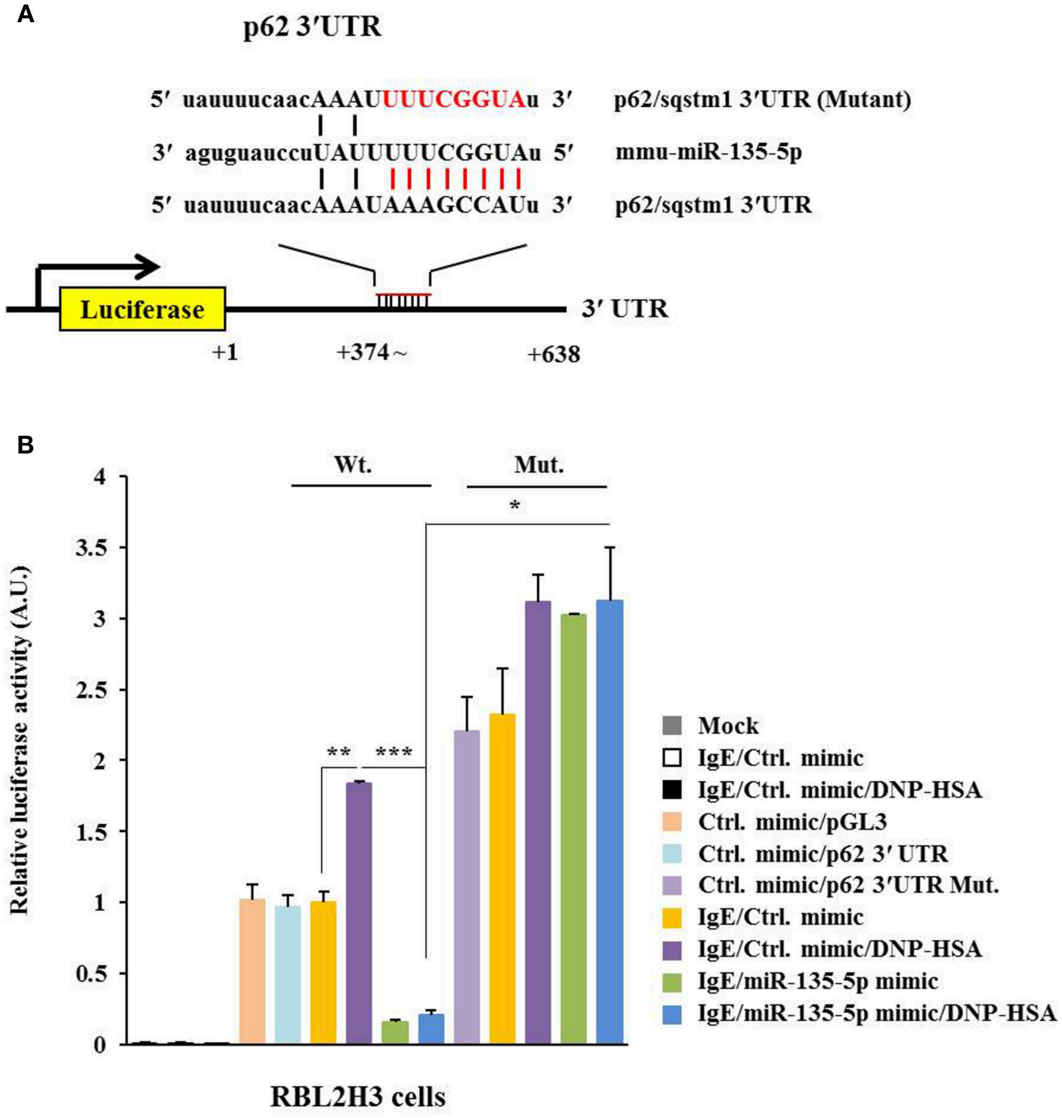
miR-135-5p directly targets p62. **(A)** Potential binding of miR-135-5p to 3′-UTR of p62. **(B)** Wild type Luc-p62-3′-UTR or mutant Luc-p62-3′-UTR was transfected along with control mimic or miR-135-5p mimic (each at 10 nM) into the indicated cell line. At 48 h after transfection, luciferase activity assays were performed. ^*^p < 0.05; ^**^p < 0.005; ^***^p < 0.005.

TargetScan analysis predicted binding of miR-135-5p to 3′-UTR of p62 (Figure 6A). Wild type and mutant 3′-UTR of p62 showed luciferase activities when they were transfected into RBL2H3 cells (Figure 6B). Antigen increased luciferase activities associated with wild type and mutant 3′-UTR of p62 (Figure 6B). MiR-135-5p mimic decreased luciferase activity associated with Luc-3′-wild type UTR of p62, but not luciferase activity associated with Luc-3′-mutant UTR of p62 in antigen-stimulated RBL2H3 cells (Figure 6B). Thus, miR-135-5p can directly regulate expression level of p62.

### MiR-135-5p Mimic Inhibits Allergic Inflammation Both *in vitro* and *in vivo*

**Figure 7:**
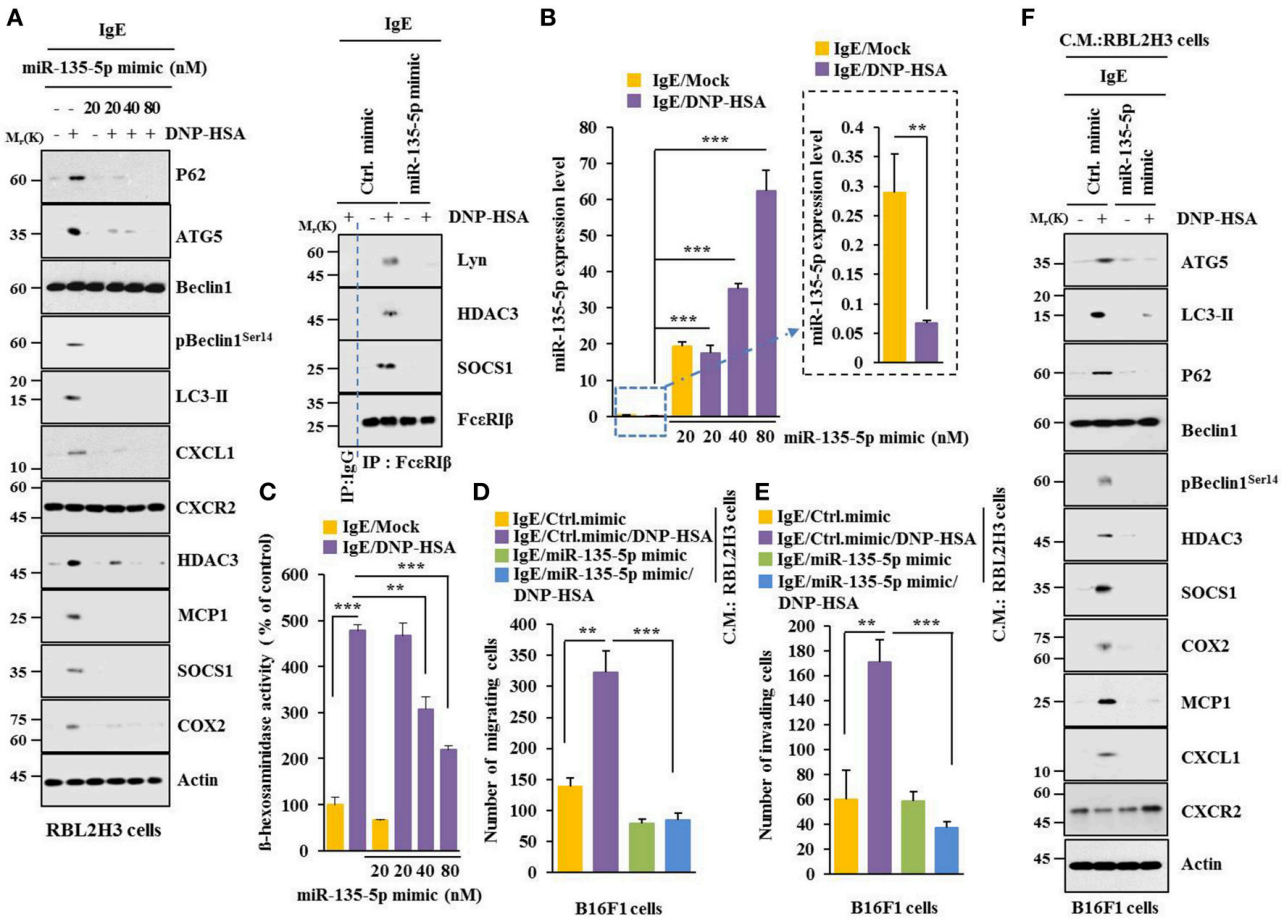
miR-135-5p mimic inhibits allergic inflammation. **(A)** RBL2H3 cells were transfected with control mimic (80 nM) or miR-135-5p mimic at indicated concentration. The next day, cells were sensitized with IgE for 24 h, stimulated with DNP-HSA for 1 h, and subjected to Immunoblot. For immunoprecipitation, RBL2H3 cells were transfected with control mimic (80 nM) or miR-135-5p mimic (20 nM). **(B)** Same as **(A)** except that qRT-PCR analysis was performed. **(C)** Same as **(A)** except that ß-hexosaminidase activity assays were performed. **(D,E)** RBL2H3 cells were transfected with indicated mimic (each at 20 nM). The next day, cells were sensitized with IgE for 24 h followed by stimulation with DNP-HSA for 1 h. The culture medium was obtained and added to B16F1 cells and incubated for 8 h followed by migration or invasion potential assays. **(F)** Same as **(E)** except that immunoblot was performed. ^**^p < 0.005; ^***^p < 0.005.

**Figure 8:**
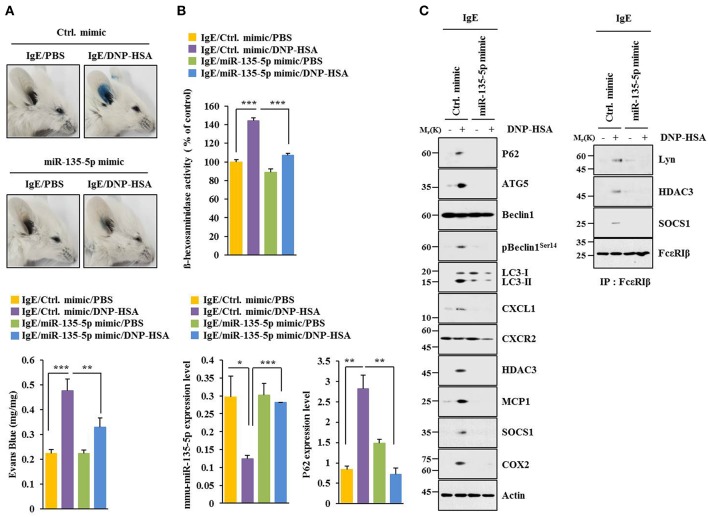
miR-135-5p mimic inhibits passive cutaneous anaphylaxis. **(A)** BALB/C mice were given an intradermal injection of IgE antibody (0.5 μg/kg) or IgG (0.5 μg/kg) along with indicated mimic (each at 100 nM). The next day, BALB/C mice were given an intravenous injection of PBS or DNP-HSA (250 μg/kg) along with 2% (v/v) Evans blue solution. One hour after the injection, the extent of vascular permeability was determined. Each experimental group consisted of four BALB/C mice. Means ± S.E. of three independent experiments are depicted. **(B,C)** Ear tissue lysate from BALB/C mouse of each experimental group was subjected to β-hexosaminidase activity assays, qRT-PCR analysis, immunoblot, and immunoprecipitation. ^*^p < 0.05; ^**^p < 0.005; ^***^p < 0.005.

MiR-135-5p mimic prevented antigen from increasing hallmarks of allergic inflammation and autophagic flux. It also prevented antigen from inducing interactions of FcεRIß with HDAC3, Lyn and SOCS1 in RBL2H3 cells (Figure 7A). Antigen decreased expression level of miR-135-5p in RBL2H3 cells (Figure 7B). MiR-135-5p mimic prevented antigen from increasing ß-hexosaminidase activity (Figure 7C). It also prevented culture medium of antigen-stimulated RBL2H3 cells from enhancing migration (Figure 7D) and invasion potential (Figure 7E) of B16F1 cells. MiR-135-5p mimic prevented culture medium of antigen-stimulated RBL2H3 cells from increasing hallmarks of allergic inflammation and autophagic flux in B16F1 cells (Figure 7F). It prevented antigen from enhancing vascular permeability (Figure 8A). It also prevented antigen from increasing ß-hexosaminidase activity (Figure 8B) and p62 expression (Figure 8B) in a mouse model of PCA. MiR-135-5p mimic also prevented antigen from increasing autophagic flux, hallmarks of allergic inflammation, and antigen from inducing interactions of FcεRIß with HDAC3, Lyn, and SOCS1 in a mouse model of PCA (Figure 8C). Thus, miR-135-5p mimic can regulate allergic inflammation both *in vitro* and *in vivo*.

### MiR-135-5p Mimic Inhibits Allergic Inflammation-Enhanced Metastatic Potential and Tumorigenic Potential of B16F1 Melanoma Cells

**Figure 9:**
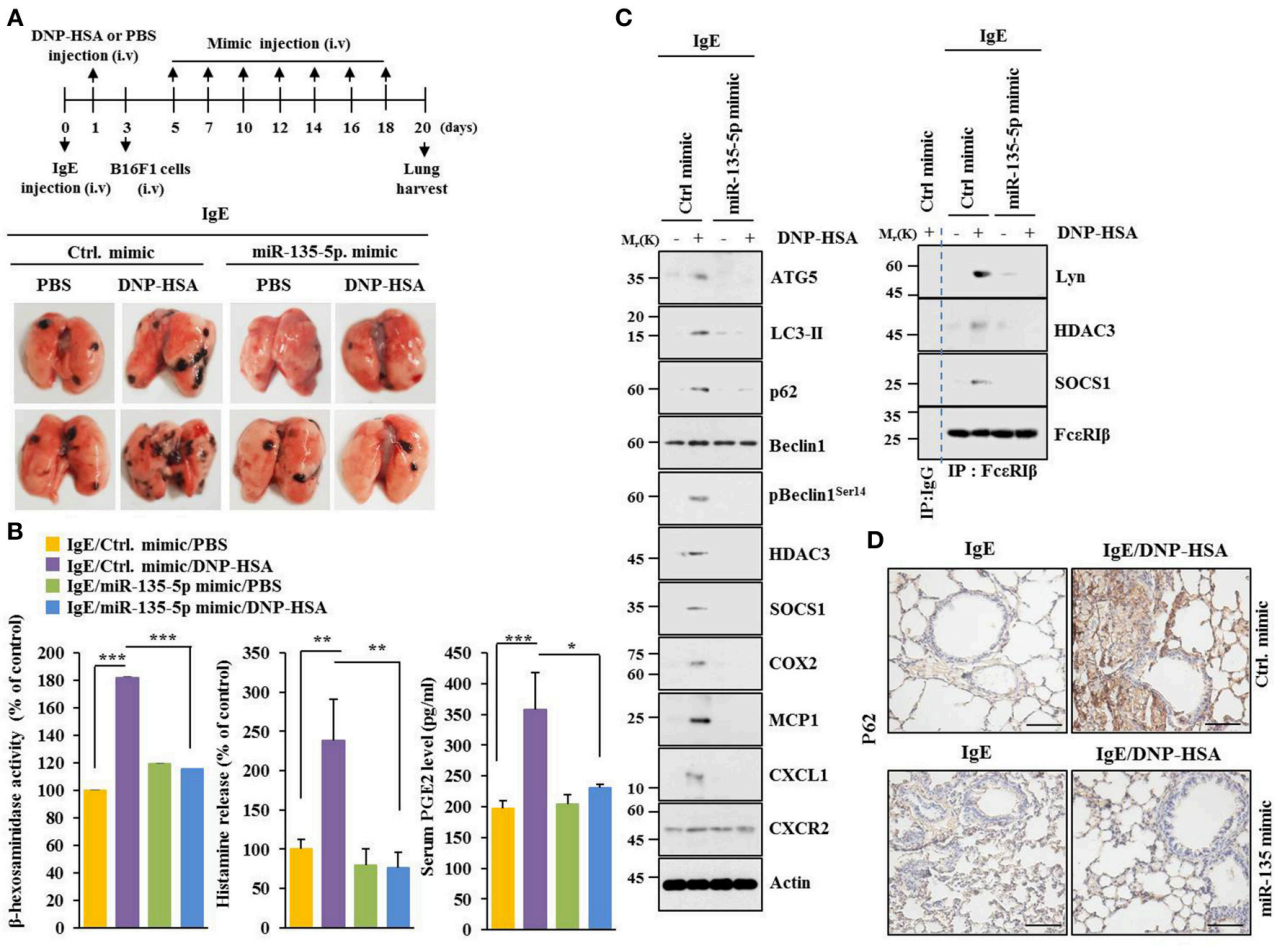
MiR-135-5p mimic inhibits passive systemic anaphylaxis (PSA)-promoted metastatic potential of B16F1 melanoma cells. **(A)** Induction of passive systemic anaphylaxis was performed as described. Each mouse received an intravenous injection of B16F1 melanoma cells (2 × 10^5^) on day 3 and intravenous injection of miR-135-5p mimic (100 nM) at indicated day. Each experimental group consisted of four BALB/C mice. **(B,C)** Tumor tissue lysates were subjected to β-hexosaminidase activity assays, immunoblot, and immunoprecipitation. Sera of BALB/C mice were employed to determine levels of histamine released and PGE2. **(D)** Immunohistochemical staining was performed. ^*^p < 0.05; ^**^p < 0.005; ^***^p < 0.005.

**Figure 10:**
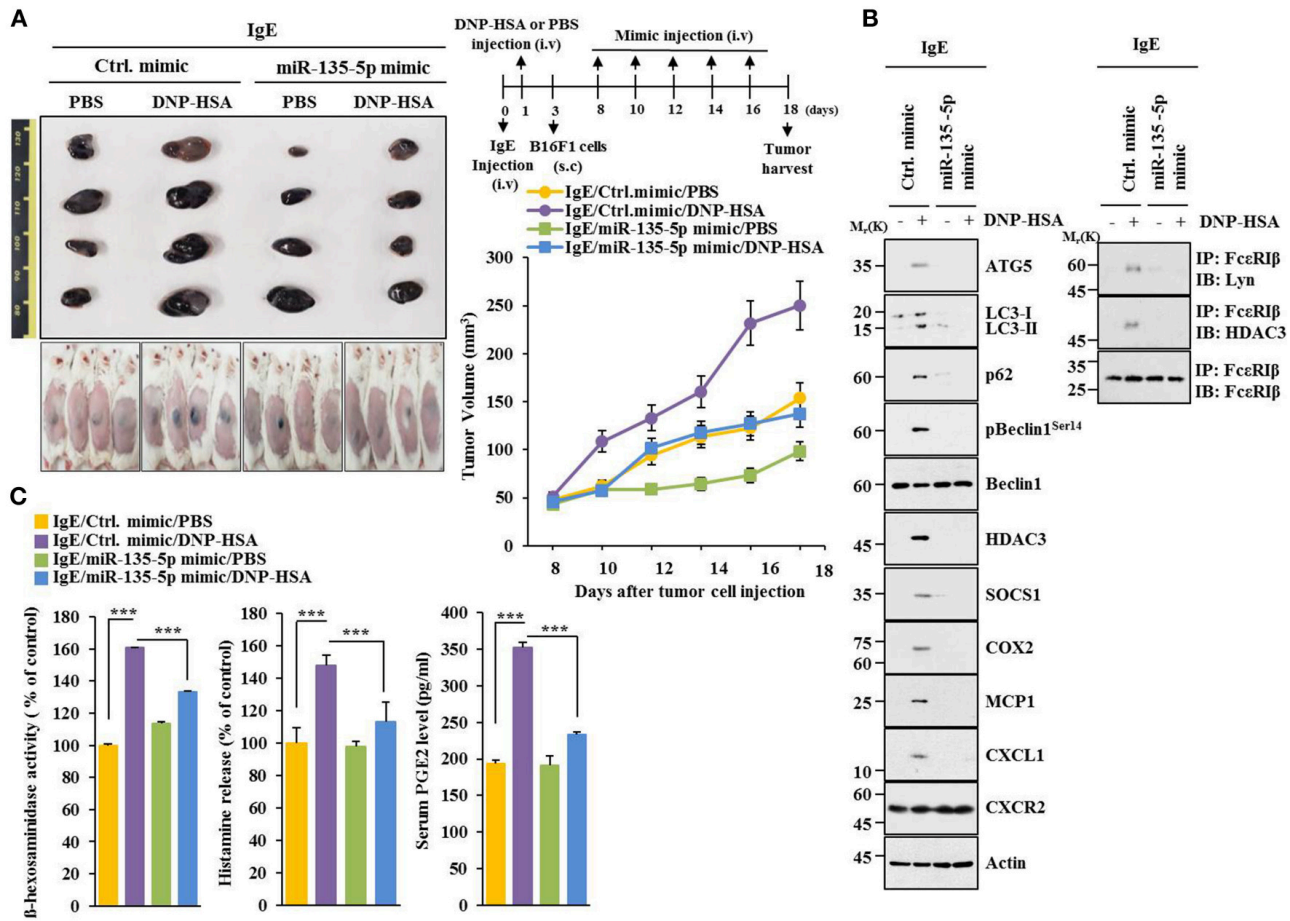
miR-135-5p mimic inhibits enhanced tumorigenic potential of B16F1 melanoma cells induced by passive systemic anaphylaxis. **(A)** Each mouse received an intravenous injection of B16F1 melanoma cells (2 × 10^5^) on day 3 and an intravenous injection of control mimic or miR-135-5p mimic (each at 100 nM) at indicated day. Each experimental group consisted of four BALB/C mice. **(B,C)** Tumor tissue lysates were subjected to immunoblot, immunoprecipitation, and β-hexosaminidase activity assays. Sera were employed to determine levels of histamine released and PGE2. ^***^p < 0.0005.

MiR-135-5p mimic prevented PSA from enhancing metastatic potential of B16F1 melanoma cells (Figure 9). It also prevented antigen from increasing β-hexosaminidase activity, amount of histamine released, and PGE2 level in BALB/C mice (Figure 9B). PGE2 is known to contribute to the development of asthma by promoting IgE production (28). MiR-135-5p mimic prevented antigen from increasing hallmarks of allergic inflammation and autophagic flux. It also prevented antigen from inducing interactions of FcεRIβ with HDAC3 and Lyn (Figure 9C). Immunohistochemical staining showed that miR-135-5p mimic prevented antigen from increasing expression level of p62 (Figure 9D). MiR-135-5p mimic prevented PSA from enhancing tumorigenic potential of B16F1 melanoma cells (Figure 10A). MiR-135-5p mimic prevented antigen from increasing hallmarks of allergic inflammation and autophagic flux. It prevented antigen from inducing interactions of FcεRIβ with HDAC3 and Lyn in tumor tissues (Figure 10B). It also prevented antigen from increasing β-hexosaminidase activity, the amount of histamine released, and PGE2 level (Figure 10C). Thus, miR-135-5p mimic can inhibit allergic inflammation- enhanced metastatic potential and tumorigenic potential of B16F1 melanoma cells.

### Extracellular Vesicles Are Necessary for Cellular Interactions During Allergic Inflammation

**Figure 11:**
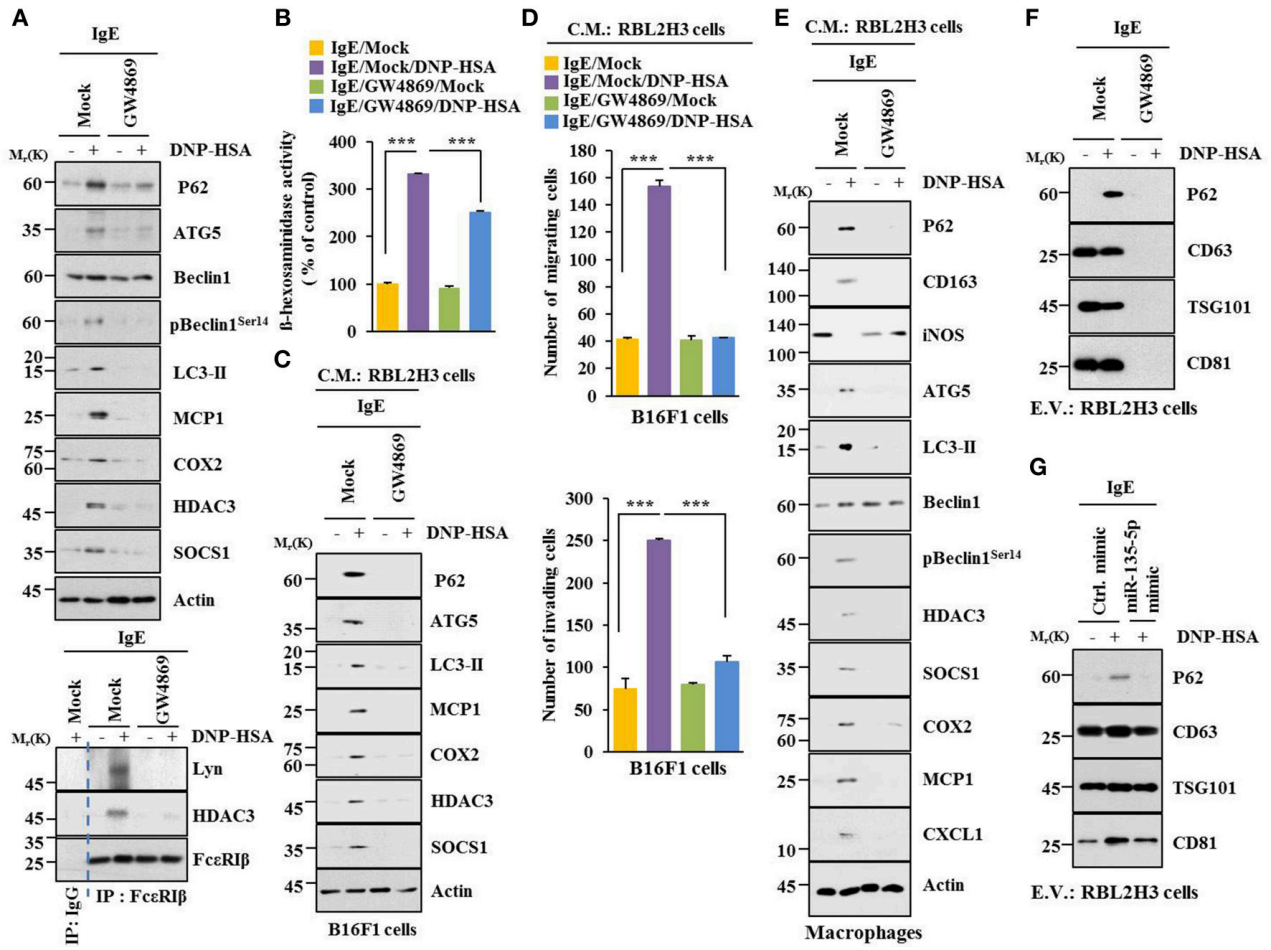
Extracellular vesicles are necessary for cellular interactions in allergic inflammation. **(A,B)** IgE-sensitized RBL2H3 cells were treated without or with GW4869 (10 μM) for 24 h followed by stimulation with DNP-HSA for 1 h. Immunoblot, immunoprecipitation and ß-hexosaminidase activity assays were performed. **(C)** Culture medium of RBL2H3 cells was added to B16F1 cells and incubated for 8 h followed by immunoblot. **(D)** Same as **(C)** except that migration and invasion potentials of B16F1 cells were determined. **(E)** Same as **(C)** except that culture medium was added to lung macrophages. **(F)** Extracellular vesicles isolated from antigen-stimulated RBL2H3 cells without or with G4869 treatment were subjected to immunoblot. **(G)** RBL2H3 cells were transfected with indicated mimic (each at 20 nM). The next day, cells were sensitized with IgE for 24 h followed by stimulation with DNP-HSA. Extracellular vesicles were isolated and subjected to immunoblot. ^***^p < 0.0005.

Extracellular vesicles of multiple myeloma (MM) cells can stimulate secretion of cytokines, such as CXCL1, MCP1, IL6, IL-, IP-10, and CCL5 in mesenchymal stromal cells (MSCs) to promote MM cell growth and migration (29). GW4869, an inhibitor of extracellular vesicles formation, decreased hallmarks of allergic inflammation and autophagic flux. It also inhibited interactions of FcεRIβ with HDAC3 and Lyn in antigen-stimulated RBL2H3 cells (Figure 11A). It prevented antigen from increasing β-hexosaminidase activity (Figure 11B). GW4869 prevented culture medium of antigen-stimulated RBL2H3 cells from increasing hallmarks of allergic inflammation and autophagic flux (Figure 11C) or enhancing migration and invasion potential of B16F1 cells (Figure 11D). GW4869 prevented culture medium of antigen-stimulated RBL2H3 cells from regulating expression levels of CD163 and iNOS, hallmarks of allergic inflammation, and autophagic flux in lung macrophages (Figure 11E). GW4869 prevented antigen from inducing expression level of p62 in extracellular vesicles of RBL2H3 cells (Figure 11F). MiR-135-5p mimic decreased expression level of extracellular vesicular p62 in antigen-stimulated RBL2H3 cells (Figure 11G). When culture medium of antigen-stimulated RBL2H3 cells was added to lung macrophages, it increased expression level of CD163, but decreased expression level of iNOS, in the absence of GW4869 (Figure S4A). Pellet fraction of growth medium of RBL2H3 cells showed extracellular vesicles (Figure S4B). Thus, extracellular vesicles can mediate cellular interactions during allergic inflammation.

### Extracellular Vesicles Are Necessary for Anaphylaxis

GW4869 prevented antigen from decreasing rectal temperatures (Figure S5A). It also prevented antigen from increasing ß-hexosaminidase activity, the amount of histamine released, and PGE2 level in a mouse model of PSA (Figure S5B). GW4869 prevented antigen from increasing hallmarks of allergic inflammation and autophagic flux. It also prevented antigen from inducing interactions of FcεRIß with HDAC3, Lyn, and SOCS1 (Figure S5C). GW4869 prevented antigen from increasing expression levels of G-CSF and MCP1 in the sera of BALB/C mouse model of PSA (Figure S5D). GW4869 prevented antigen from increasing vascular permeability (Figure S6A), autophagic flux, and hallmarks of allergic inflammation (Figure S6B). It also prevented antigen from inducing interactions of FcεRIß with HDAC3, SOCS1, and Lyn (Figure S6B) in a mouse model of PCA. GW4869 also prevented antigen from increasing ß-hexosaminidase activity (Figure S6C). Thus, extracellular vesicles can mediate anaphylaxis *in vivo*.

### Extracellular Vesicles Contain p62, Shuttle Between Cells and Induce Features of Allergic Inflammation

**Figure 12:**
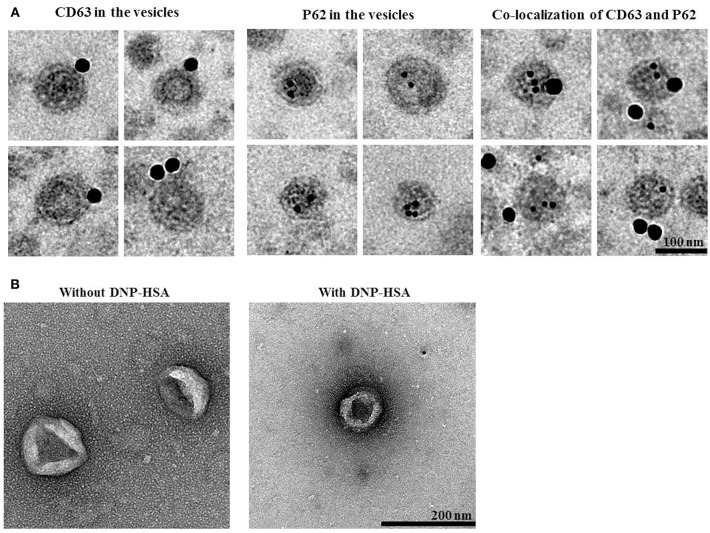
P62 is present in extracellular vesicles of antigen-stimulated RBL2H3 cells. **(A)** General appearances of isolated extracellular vesicles and immuno-gold staining images using anti-CD63, a known membrane marker for the extracellular vesicles, and anti-p62 antibodies. Twenty-five and 10 nm gold particles indicate the localization of CD63 (outer membrane of the vesicles) and p62, respectively. Note that p62 is shown to locate in the lumen of the vesicles. **(B)** Extracellular vesicles isolated from un-stimulated RBL2H3 cells or antigen-stimulated RBL2H3 cells were visualized by negative staining electron microscopy. One hundred and 200 nm scale bars applied to the montages **(A)** and the fields **(B)**, respectively.

We next examined whether p62 exists in extracellular vesicles, by using immunogold-staining electron microscopy. Immunogold-conjugated p62 antibody was used to determine the location of P62 in the isolated vesicles, and P62 was detected in the lumen of the vesicle, whereas CD63, a known membrane marker of extracellular vesicles, was detected in the outer membrane of the vesicles (Figure 12A). This observation was confirmed by co-immunogold staining of p62 (as shown by 10 nm golds located in the inner of the vesicles) and CD63 (as shown by 25 nm golds located in the outer membrane of the vesicles). Visualized extracellular vesicles under negative staining electron microscopy demonstrated the existence of the vesicles in RBL2H3 cells regardless of antigen stimulation (Figure 12B).

PKH67-labeled extracellular vesicles were added to RBL2H3 cells to examine whether extracellular vesicles could shuttle between cells. Green fluorescence was observed in RBL2H3 cells that took up PKH67-labeled extracellular vesicles of un-stimulated RBL2H3 cells and antigen-stimulated RBL2H3 cells (Figure S7A). However, fluorescence was not observed in RBL2H3 cells that took up un-labeled extracellular vesicles of antigen-stimulated RBL2H3 cells (Figure S7A). Extracellular vesicles of antigen-stimulated RBL2H3 cells increased levels of histamine released and PGE2 in RBL2H3 cells (Figure S7B). Thus, extracellular vesicles can shuttle between cells and induce features of allergic inflammation.

### Extracellular Vesicles Promote Features of Allergic Inflammation in a p62-Dependent Manner

**Figure 13:**
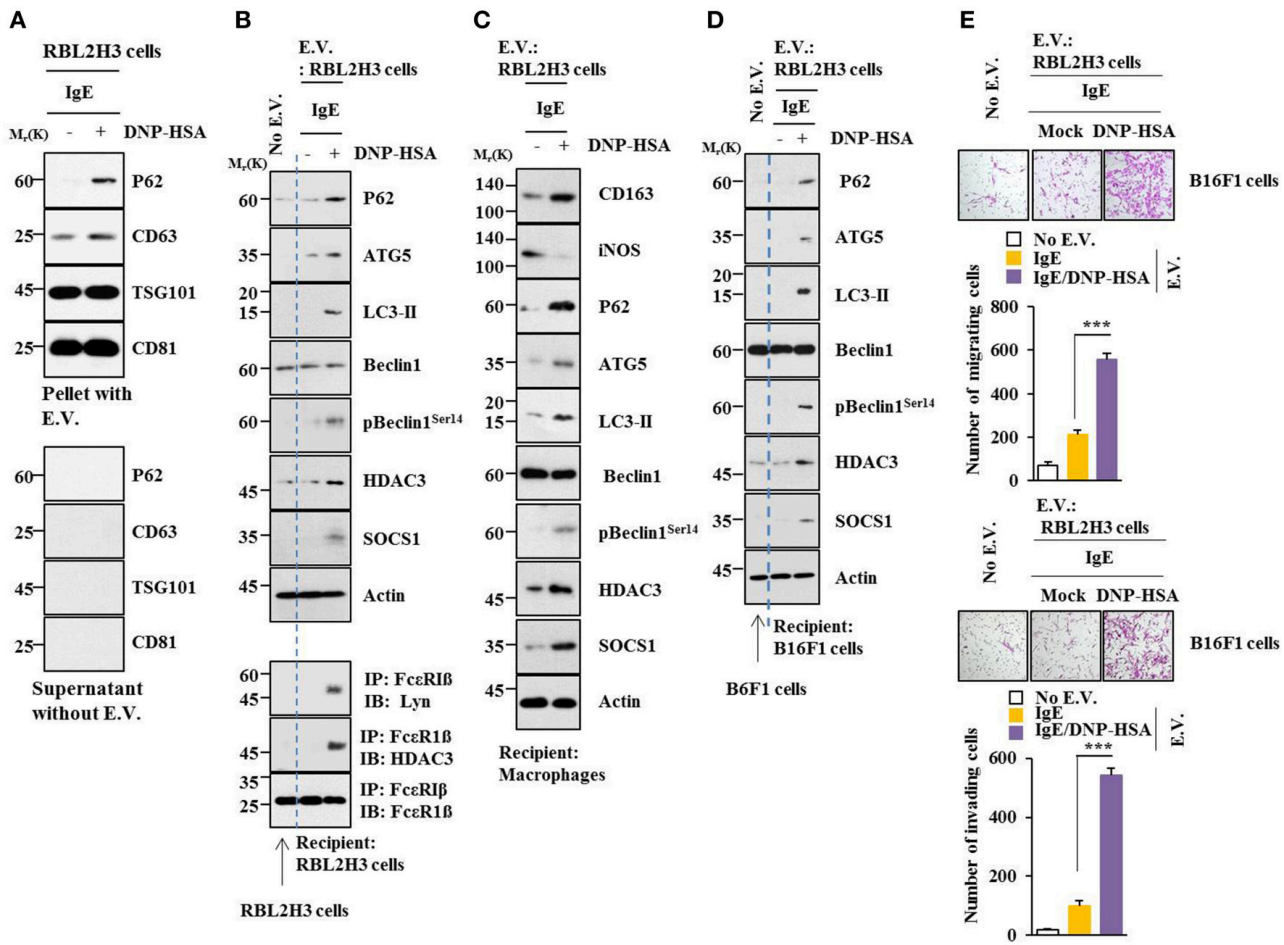
Extracellular vesicles promote features of allergic inflammation**. (A)** Extracellular vesicles were isolated from growth medium of RBL2H3 cells without or with antigen stimulation for 1 h. Extracellular vesicular proteins were subjected to immunoblot. E.V. denotes extracellular vesicles. **(B,C)** Extracellular vesicles (20 μg) isolated from RBL2H3 cells without or with antigen stimulation for 1 h were added to RBL2H3 cells or lung macrophages and incubated for 24 h followed by immunoblot and immunoprecipitation. No extracellular vesicles denote immunoblot and immunoprecipitation of RBL2H3 cells. **(D)** Same as **(B)** except that extracellular vesicles were added to B16F1 cells. No extracellular vesicles denote immunoblot of B16F1 cells without extracellular vesicles treatment. **(E)** Same as **(D)** except that migration and invasion potential assays were performed. No extracellular vesicles denote migration or invasion potential of B16F1 cells without extracellular vesicles treatment. ^***^p < 0.0005.

Using GW4869, an inhibitor of extracellular vesicles formation, results showed that extracellular vesicles played a role in anaphylaxis (Figure S5A). Therefore, direct effect of extracellular vesicles on allergic inflammation was examined. Markers of extracellular vesicles, such as CD63, TSG101 and CD81 were found in the pellet fraction of growth medium, but not in the supernatant fraction of growth medium, of RBL2H3 cells (Figure 13A). P62 was also present in the pellet fraction of growth medium, but not in the supernatant fraction of growth medium, of antigen-stimulated RBL2H3 cells (Figure 13A). Extracellular vesicles isolated from antigen-stimulated RBL2H3 cells increased hallmarks of allergic inflammation and autophagic flux. They also induced interactions of FcεRIß with HDAC3 and Lyn in unstimulated RBL2H3 cells (Figure 13B). Extracellular vesicles isolated from antigen-stimulated RBL2H3 cells increased CD163 and hallmarks of allergic inflammation and autophagic flux, but decreased expression of iNOS in macrophages (Figure 13C). Extracellular vesicles isolated from antigen-stimulated RBL2H3 cells increased expression of p62, hallmarks of allergic inflammation, and autophagic flux in B16F1 cells (Figure 13D). They also enhanced migration and invasion potentials of B16F1 cells (Figure 13E). Extracellular vesicles of antigen-stimulated RBL2H3 cells contained p62 (Figure S8A). They increased autophagic flux and CD163, but decreased expression of iNOS in a p62-dependent manner in lung macrophages (Figure S8B). Extracellular vesicles of antigen-stimulated RBL2H3 cells increased autophagic flux in RBL2H3 cells (Figure S8C). They also induced interactions of FcεRIß with HDAC3 and Lyn in a p62-dependent manner (Figure S8C). Extracellular vesicles of antigen-stimulated RBL2H3 cells also increased levels of histamine released and PGE2 in RBL2H3 cells in a p62-dependent manner (Figure S8D). Extracellular vesicles of antigen-stimulated RBL2H3 cells increased autophagic flux (Figure S8E) and enhanced migration and invasion potentials of B16F1 cells in a p62-dependent manner (Figure S8F). Effect of p62 on extracellular vesicles -mediated cellular interactions was further investigated. For this, we employed extracellular vesicles isolated from sera of PSA-activated BALB/C mouse (Figure S9A). Extracellular vesicles isolated from serum of PSA-activated BALB/C mouse showed expression of p62 (Figure S9B). Serum of each mouse of each experimental group in the mouse model of PSA showed extracellular vesicles (Figure S9C). Extracellular vesicles increased autophagic flux and hallmarks of allergic inflammation (Figure S9D) and induced interactions of FcεRIß with HDAC3, Lyn, and SOCS1 in unstimulated RBL2H3 cells (Figure S9E). They also enhanced migration and invasion potentials of B16F1 cells in a p62-dependent manner (Figure S9F). Thus, p62 is necessary for extracellular vesicles -mediated cellular interactions during allergic inflammation.

## Discussion

Neutrophil autophagy enhances asthma severity by damaging airway epithelium and triggering inflammatory responses (30). TLR2 confers a pivotal role in allergic airway inflammation via regulating PI3K/Akt signaling pathway-related autophagy in mice (31). Positive correlation between gene expression patterns of ATG5 and COL5A1 suggests that dysregulated autophagy may contribute to subepithelial fibrosis in airways of refractory asthmatic individuals (32). Expression of beclin-1 was upregulated in airways of patients with asthma and OVA-challenged mice, accompanied by airway EMT and remodeling (33). More autophagosomes are found in patients with asthma and OVA-challenged mice compared with healthy controls (33). Autophagy is closely correlated with the severity of asthma through eosinophilic inflammation (34). These reports suggest a close relationship between autophagy and allergic inflammation.

p62 was increased during allergic inflammation (Figure 1A). It regulated hallmarks of allergic inflammation and autophagic flux (Figure 1B). We also showed that allergic inflammation was accompanied by enhanced autophagosome formation (Figure S1). It will be interesting to examine the effect of p62 on autophagosome formation during allergic inflammation in future studies. It will also be necessary to identify molecule regulated by p62. 3-MA, an inhibitor of autophagy, prevented antigen from increasing expression of p62 and hallmarks of allergic inflammation in RBL2H3 cells (Figure S2A). 3-MA also negatively regulated PCA (Figure S2D). This indicates a role of autophagy in allergic inflammation. The role of autophagy in anaphylaxis has not been reported yet.

Antigen stimulation increased expression of HDAC3 in RBL2H3 cells (Figure 1A). Role of HDAC3 in allergic inflammation has been reported (11, 26). MiR-384 and HDAC3 can form a negative feedback loop to regulate allergic inflammation and cellular interactions during allergic inflammation (26). MiR-384, a negative regulator of HDAC3, can reduce augmentation of Beclin1-dependent autophagy of airway smooth muscle cells (35). Hdac3-deficient iNKT cells showed less Cyto-ID staining and lower LC3A/B expression, indicating reduced autophagy (36). HDAC3 may regulate autophagic flux during allergic inflammation. Further studies are needed to identify miRNAs that regulate expression of HDAC3 during allergic inflammation.

COX2 is known to be an asthma-associated gene (37). Allergic inflammation increased expression of COX2 in RBL2H3 cells (Figure S2E). COX2 and miR-26 can form a negative feedback loop and regulate allergic inflammation and cellular interactions during allergic inflammation (18). COX2 overexpression induced by the ATF4 ER stress pathway contributes to Lupus Nephritis-induced kidney autophagy and injury (38). It is probable that miR-26/COX2 axis may regulate autophagic flux during allergic inflammation.

TargetScan analysis predicted binding of miR-181a/-218/-122a to the 3′UTR of p62 (personal observation). MiR-181a/-218 can form a negative feedback loop with TGaseII and regulate allergic inflammation (25). Allergic inflammation increased expression of TGaseII in RBL2H3 cells (Figure 1A). Under stress, TGaseII mediates enhanced autophagy to promote Mantle cell Lymphoma (MCL) (39). Autophagy product ATG5 involved in autophagosome elongation can positively regulate TGase II/NF-κB/IL6 signaling (39). MiR-181a mimic prevented antigen from increasing expression levels of TGaseII and p62 in RBL2H3 cells while miR-181a inhibitor increased expression levels of TGaseII and p62 in an antigen-independent manner in RBL2H3 cells (personal observations). It will be necessary to identify miRNAs that can regulate expression of TGaseII in the future.

Increased level of CXCL1 has been reported in a mouse model of allergic rhinitis (40). Allergic inflammation increased expression of CXCL1 in RBL2H3 cells (Figure 7A). MiR-135-5p mimic prevented antigen from increasing expression of CXCL1 in RBL2H3 cells (Figure 7A). Neutrophilic inflammation, a hallmark of allergic asthma, is mediated by CXCR2, a receptor of CXCL1 (41). CXCR2 can enhance neutrophilic inflammation and exacerbate IL-33-induced airway hyper responsiveness (41). Neutrophilic asthma in STAT6^−/−^ mice that are steroid resistant is accompanied by elevated lung levels of TNF-α, CXCL1, CXCL2, and CXCL5 (42). Mast cell-derived CXCL1 mediates the protumorigenic role of mast cells (43). It is probable that CXCL1 can mediate cellular interactions during allergic inflammation. It would be interesting to examine signaling pathways of CXCL1-CXCR2 axis for better understanding of p62-promoted allergic inflammation. It is also important to identify cytokines/miRNAs that can serve as targets of CXCL1.

Intravitreal application of miR-135 facilitates retinal ganglion cell (RGC) axon regeneration after optic nerve injury in adult mice in part by repressing KLF4 (44). Lack of Kruppel-like factor 4 (KLF4) expression in monocytes and lung epithelial cells decreases Th2 cytokines in mice and airway hyper responsiveness (AHR) (45). Endogenous KLF4 can bind to promoter regions of p62 gene while upregulation of KLF4 induces expression of p62 (46). Thus, KLF4 might mediate allergic inflammation both *in vitro* and *in vivo* in association with autophagic processes by regulating expression level of p62. TargetScan analysis predicted that miR-135-5p was a negative regulator of p62 (i.e., miR-135-5p directly regulated expression of p62) (Figure 6). MiR-135-5p mimic had a negative regulatory role in *in vitro* allergic inflammation (Figures 7A,C). MiR-135-5p mimic negatively regulated cellular interactions during allergic inflammation (Figures 7D,F). It will be necessary to identify cytokines that are regulated by miR-135-5p mimic. These cytokines may mediate cellular interactions during allergic inflammation. MiR-135-5p mimic can inhibit PCA (Figures 8A,C). MiR-135mimic negatively regulated metastatic potential of cancer cells enhanced by PSA (Figures 9A,B).

MiR-135-5p targets Smad5, a key transducer of the BMP2 osteogenic signal, and inhibits differentiation of osteoprogenitors (47). BMP2 is involved in allergic airway inflammation induced by house dust mite (48). Mast cells-derived histamine induces BMP-2 expression in human coronary artery endothelial cells (49). Thus, BMP2 might act as a target of miR-135-5p and mediates anaphylaxis in conjunction with autophagy.

Extracellular vesicles regulate anti-cancer drug-sensitivity by promoting autophagy (50). Human umbilical cord mesenchymal stem cells (MSC)-derived extracellular vesicles (hucMSC-Ex) can promote autophagy to prevent cisplatin-induced renal injury (51). Extracellular vesicles of mesenchymal stem cells activate regulatory T cells to suppress asthma (52). These reports suggest that extracellular vesicles have roles in allergic inflammation. GW4869, an inhibitor of extracellular vesicles formation, negatively regulated PSA (Figure S5A) and PCA (Figure S6A). Thus, extracellular vesicles can mediate anaphylaxis.

Extracellular vesicles isolated from infected macrophages can stimulate secretion of cytokines, such as RANTES, IL-1ra, MIP-2, CXCL1, MCP1, sICAM-1, and G-CSF (53). Thus, extracellular vesicles might mediate cellular interactions during allergic inflammation. GW4869, an inhibitor of extracellular vesicles formation, prevented antigen from increasing expression of hallmarks of allergic inflammation and autophagic flux in RBL2H3 cells (Figure 11A). GW4869 prevented culture medium of antigen-stimulated RBL2H3 cells from enhancing invasion and migration potentials of B16F1 cells (Figure 11D). These results indicate a role of extracellular vesicles in allergic inflammation.

GW4869 prevented antigen from increasing expression levels of MCP1 and CXCL1 in a mouse model of PSA (Figure S5C). GW4869 prevented antigen from stimulating secretion of MCP1 in serum of PSA-activated BALB/C mouse (Figure S5D). MCP1 in B16F1 cells was increased by culture medium of antigen-stimulated mast cells in a p62-dependent manner (Figure 5E). Thus, MCP1 and CXCL1 might mediate cellular interactions during allergic inflammation. It will be necessary to examine the presence of MCP1 and/or CXCL1 in extracellular vesicles of activated immune cells, such as mast cells and macrophages, during allergic inflammation. We showed the presence of p62 in extracellular vesicles of antigen-stimulated RBL2H3 cells (Figures 12A, 13A). Extracellular vesicles of antigen-stimulated RBL2H3 cells activated macrophages (Figure 13C) and enhanced invasion and migration potentials of B16F1 cells (Figure 13D). These extracellular vesicles might induce features of allergic inflammation in antigen-independent manner. It will be necessary to further identify miRNAs and cytokines present within extracellular vesicles of antigen-stimulated RBL2H3 cells. It will also be necessary to identify molecules regulated by these extracellular vesicles.

MiRNA array analysis was performed to identify miRNAs regulated by p62. Our results showed that miR-154-5p and miR-31-5p were increased in RBL2H3 cells by antigen stimulation in a p62-dependent manner (personal observations). Increased expression level of miR-154-5p was also observed in extracellular vesicles of antigen-stimulated RBL2H3 cells (data not shown). Our results showed that miR-154-5p was necessary for allergic inflammation both *in vitro* and *in vivo* (data not shown). Promoter sequences of miR-154-5p and miR-31-5p contain binding sites for HDAC2, SP1, and YY (personal observations). Therefore, SP1 and YY1 might directly increase expression levels of miR-154-5p and miR-31-5p. TargetScan analysis predicted binding of miR-154-5p to the 3′-UTR of SOCS5 and binding of miR-31-5p to the 3′-UTR of oxidative stress responsive-1 (Oxsr-1). SOCS5 can reduce JAK2 phosphorylation (54). JAK2 is necessary for allergic inflammation (21). Therefore, SOCS5 might be a negative regulator of allergic inflammation. MiR-31-5p is a candidate master regulator of genes associated with neutrophil recruitment. It targets Oxsr-1 (55). It would be interesting to examine whether Oxsr-1 is a negative regulator of allergic inflammation in the future.

In summary, we showed novel roles of miR-135-5p-p62 axis in allergic inflammation in conjunction with autophagic flux. It would be necessary to further identify extracellular vesicular cytokines and miRNAs to better understand p62-mediated allergic inflammation and cellular interactions during allergic inflammation.

## Author Contributions

DJ conceived the study, contributed to experimental design, and wrote paper. MK performed *in vitro* and *in vivo* experiments. YP, YKw, and YKi contributed with *in vivo* experimental data. JM contributed with electron microscopic observations of autophagosomes. HJ, H-UK, and MJ contributed with extracellular vesicles isolation and electron microscopic observations of extracellular vesicles. JB contributed with luciferase constructs.

### Conflict of Interest Statement

The authors declare that the research was conducted in the absence of any commercial or financial relationships that could be construed as a potential conflict of interest.
