# Supplementary material for: MiR-135-5p-p62 Axis Regulates Autophagic Flux, Tumorigenic Potential, and Cellular Interactions Mediated by Extracellular Vesicles During Allergic Inflammation
Source: Front Immunol. 2019 Apr 5;10:738. doi: 10.3389/fimmu.2019.00738 (PMC6460569; doi:10.3389/fimmu.2019.00738)
Supplement: Supplementary file 1 [file Data_Sheet_1.pdf]

Supplement to

MiR-135-5p-p62 axis regulates autophagic flux, tumorigenic potential, and cellular interactions mediated by extracellular vesicles during allergic inflammation

Misun Kim, Yeongseo Park, Yoojung Kwon, Youngmi Kim, Jaehwan Byun, Myeong Seon Jeong, Han-Ul Kim, Hyun Suk Jung, Ji Young Mun & Dooil Jeoung

Figure S1.

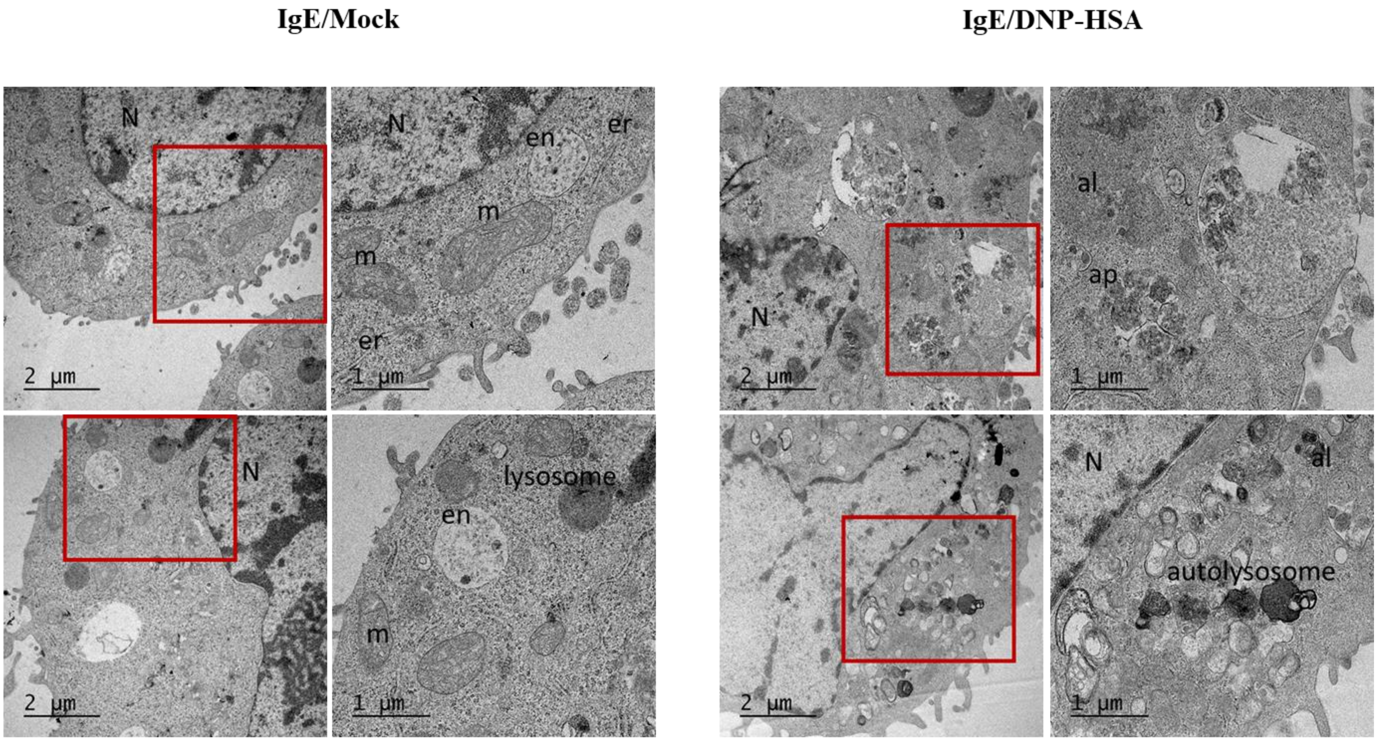

Figure S1. Antigen stimulation enhances autophagic processes. Representative electron micrograph of IgE-sensitized RBL2H3 cells stimulated without or with DNP-HSA (100 ng/ml) for 2 h. N: nucleus; en: endosome; m: mitochondria; er: endoplasmic reticulum; al: autolysosome; ap: autophagosome.

Figure S2.

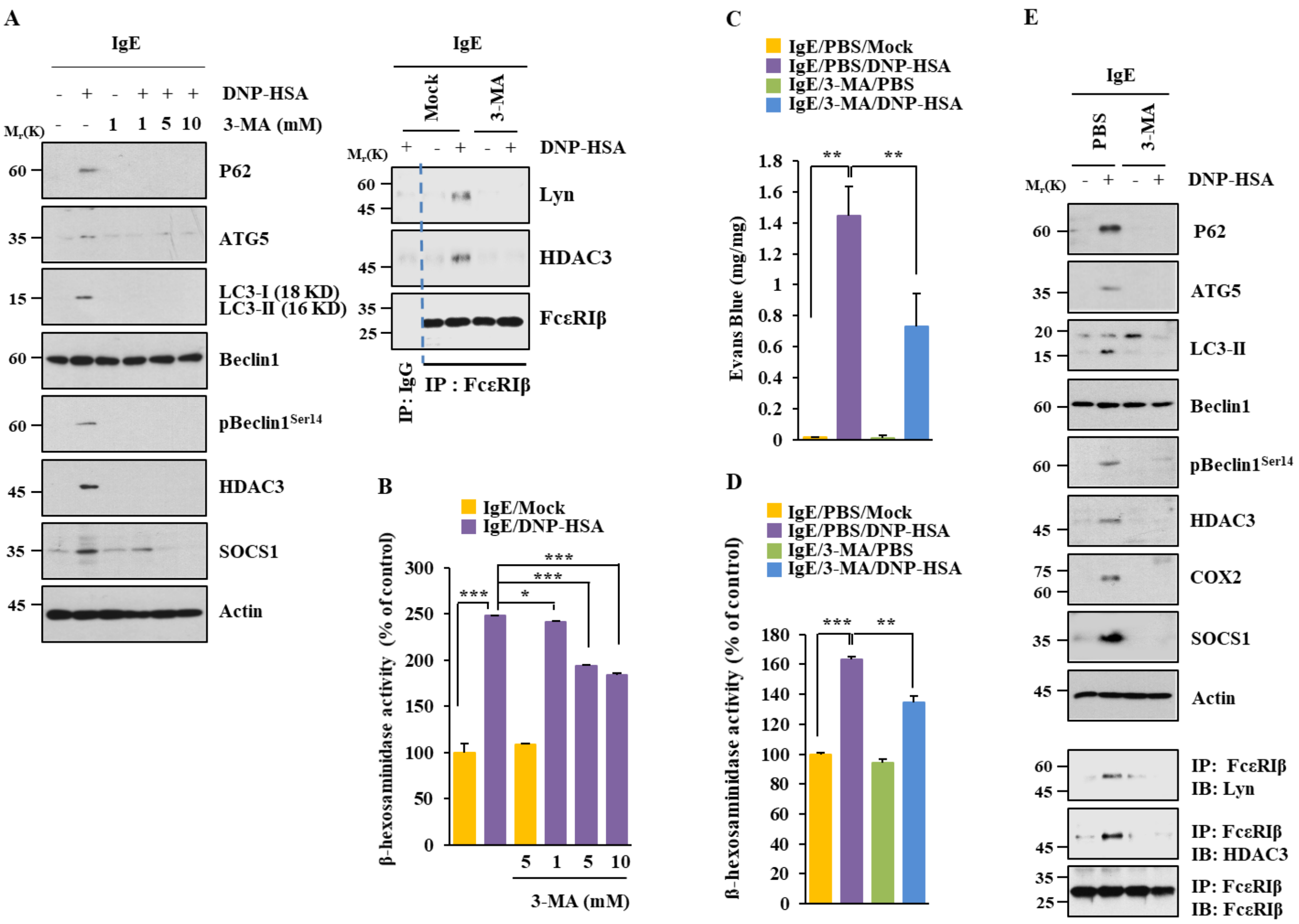

Figure S2. **3-MA, an inhibitor of autophagic processes, negatively regulates allergic inflammation *in vitro* and *in vivo*.** (A) IgE-sensitized RBL2H3 cells were treated without or with various concentrations of 3-MA for 12 h followed by stimulation with DNP-HSA for 1 h. Each blot is a representative of three independent experiments. (B) IgE-sensitized RBL2H3 cells were treated without or with various concentrations of 3-MA for 12 h followed by stimulation with DNP-HSA for 1 h. (C) BALB/C mice were given an intradermal injection of DNP-IgE (0.5 µg/kg) along without or with intravenous injection of 3-MA (200 µg/mouse). The next day, BALB/C mice were given an intravenous injection of PBS or DNP-HSA (250 µg/kg) along with 2% (v/v) Evans blue solution. Representative images from four BALB/C mice each experimental group are shown. **, p<0.005. (D) Ear tissue lysates from BALB/C mouse of each experimental group were subjected to ß-hexosaminidase activity assays. (E) Same as (D) as immunoblot and immunoprecipitation were performed.

FIGURE S3

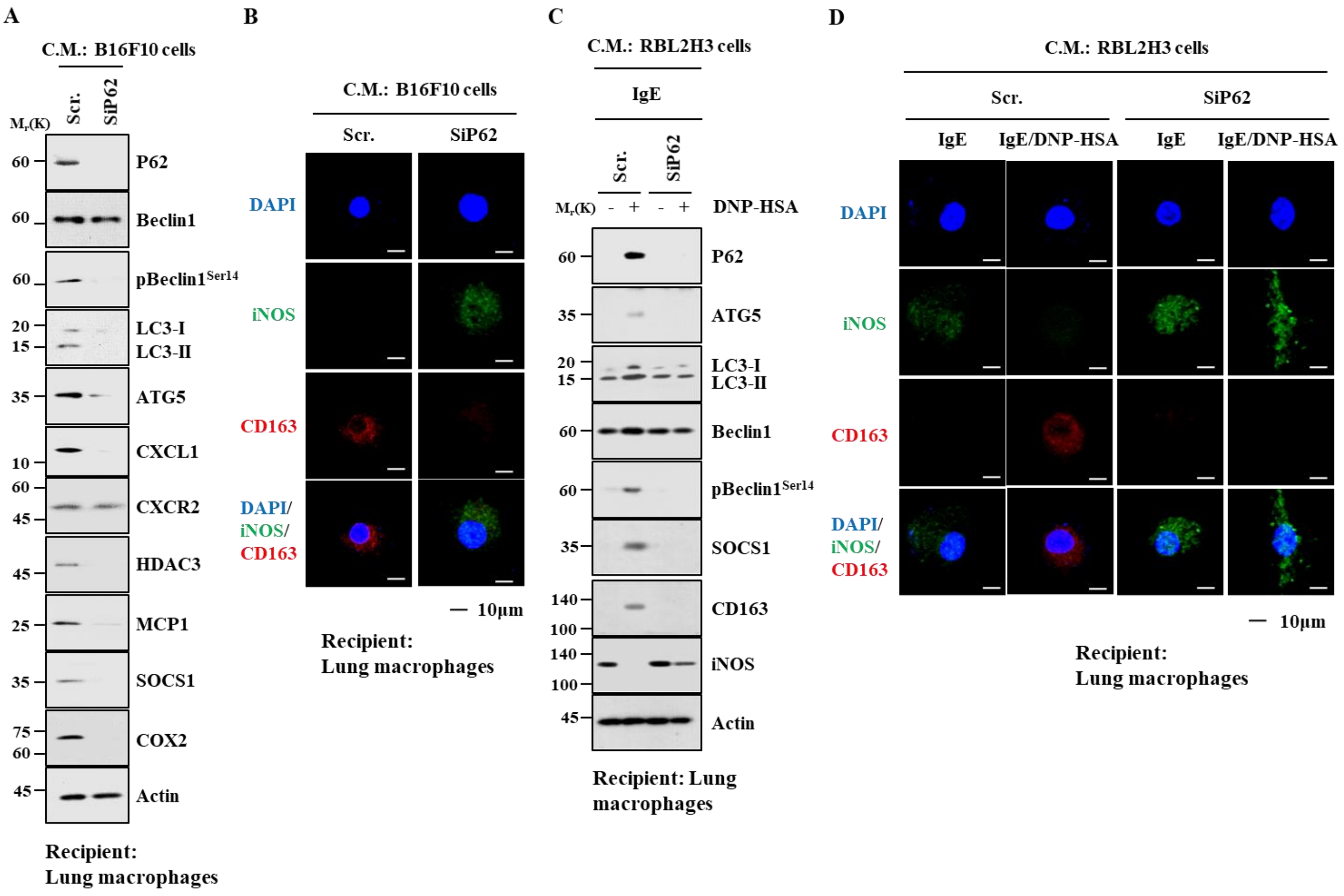

Figure S3. Tumor cells and antigen-stimulated RBL2H3 cells activate macrophages in a p62-dependent manner. (A) B16F10 cells were transfected with indicated siRNA. At 48 h after transfection, culture medium was obtained, added to lung macrophages, and incubated for 8 h followed by immunoblot. C.M. denotes culture medium. (B) same as (A) except that immunofluorescence staining was performed. (C) Culture medium of RBL2H3 cells were added to lung macrophages and immunoblot was performed. (D) Same as (C) except that immunofluorescence staining was performed.

FIGURE S4

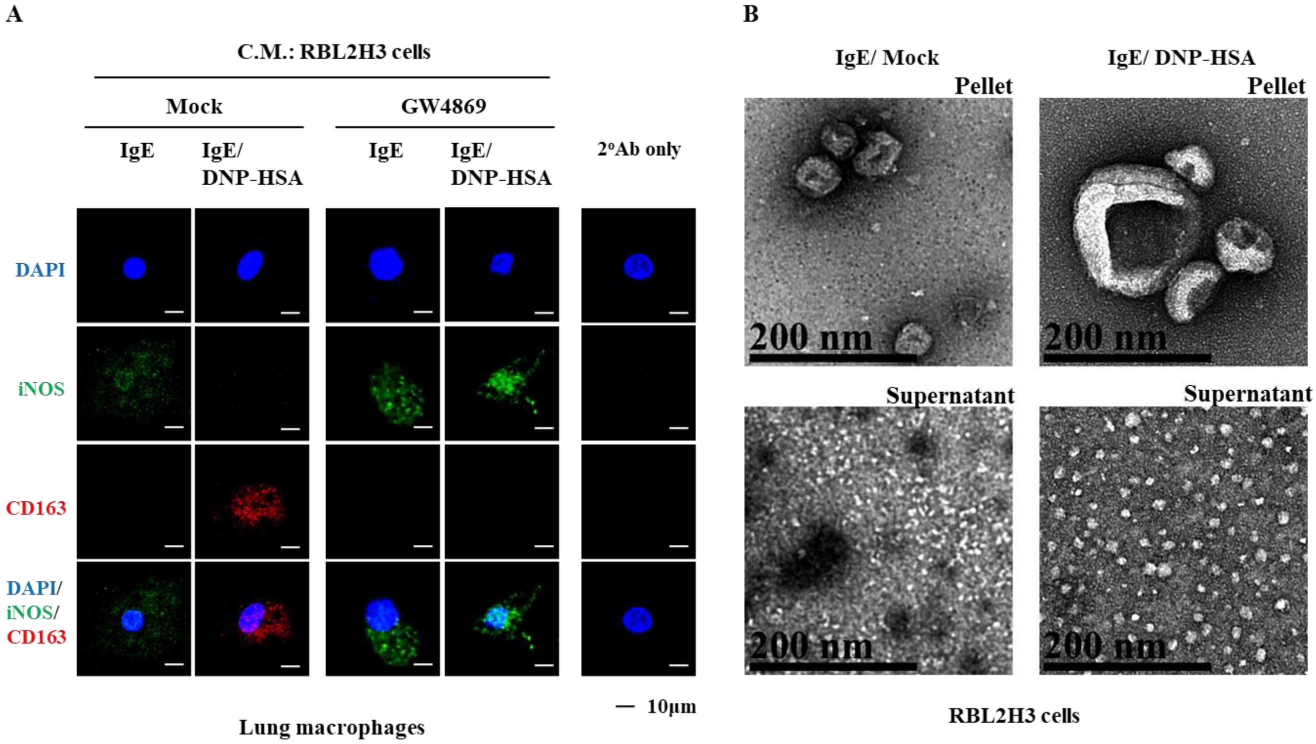

Figure S4. Extracellular vesicles are necessary for activation of macrophages. (A) IgE-sensitized RBL2H3 cells were treated without or with GW4869 (10 μM) for 24 h followed by stimulation with DNP-HSA for 1 h. C.M. was obtained and added to lug macrophages for 8 h followed by immunofluorescence staining. C.M. denotes culture medium. (B) Extracellular vesicles were isolated from RBL2H3 cells. Electron microscopic observations show extracellular vesicles in pellets.

FIGURE S5

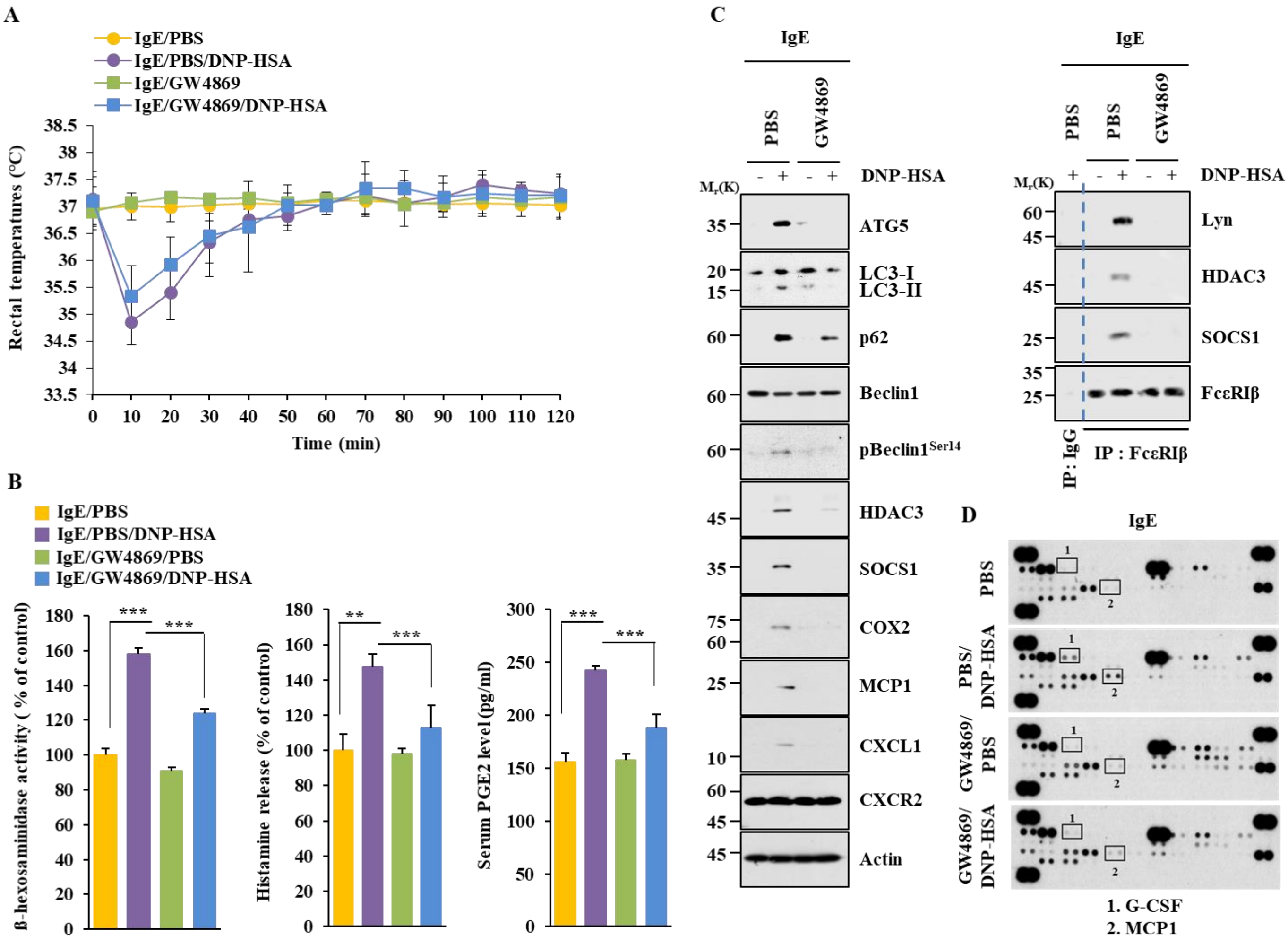

Figure S5. Extracellular vesicles are necessary for passive systemic anaphylaxis. (A) BALB/C mice were given an intravenous injection of IgE along without or with GW4869 (50 µg/mouse). The following day, BALB/C mice were intravenously injected with DNP-HSA and rectal temperatures were measured. Each experimental group consisted of five BALB/C mice. Means ± S.E. of three independent experiments are depicted. (B, C) Tissue lysates were subjected to β-hexosaminidase activity assay, immunoblot, and immunoprecipitation. Sera of BALB/C mice were employed to determine the amount of histamine released and PGE2 level. (D) Serum from BALB/C mouse of each experimental group was subjected to cytokine array analysis.

FIGURE S6

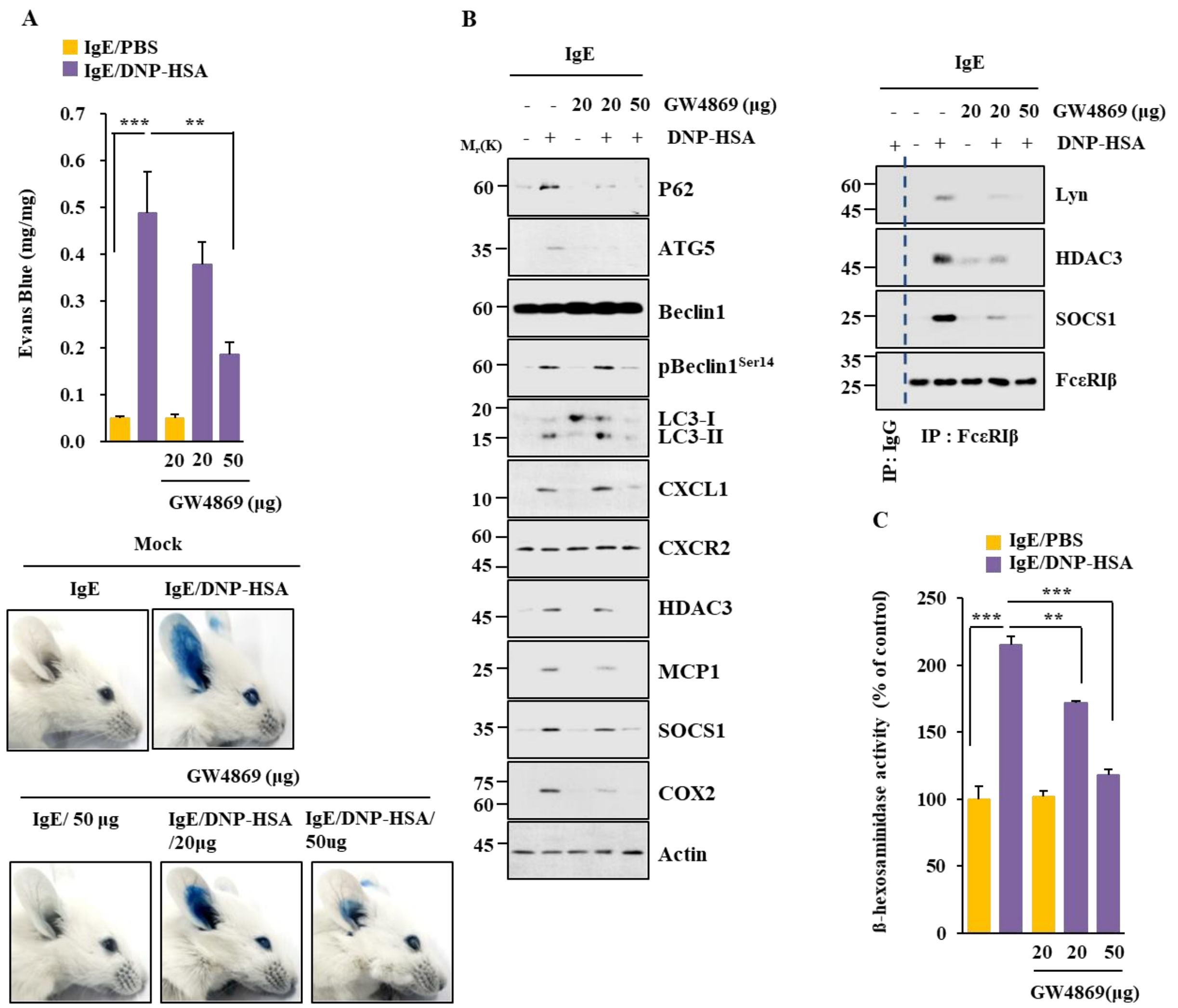

Figure S6. Extracellular vesicles are necessary for passive cutaneous anaphylaxis. (A) BALB/C mice were given an intradermal injection of IgE (0.5 μg/kg) along without or with intravenous injection of GW4869 (20, 50 μg/mouse). The next day, BALB/C mice were given an intravenous injection of PBS or DNP-HSA (250 μg/kg) along with 2% (v/v) Evans blue solution. Each experimental group consisted of four BALB/C mice. (B, C) Ear tissue lysates from BALB/C mouse of each experimental group were subjected to immunoblot, immunoprecipitation, and β-hexosaminidase activity assays.

FIGURE S7

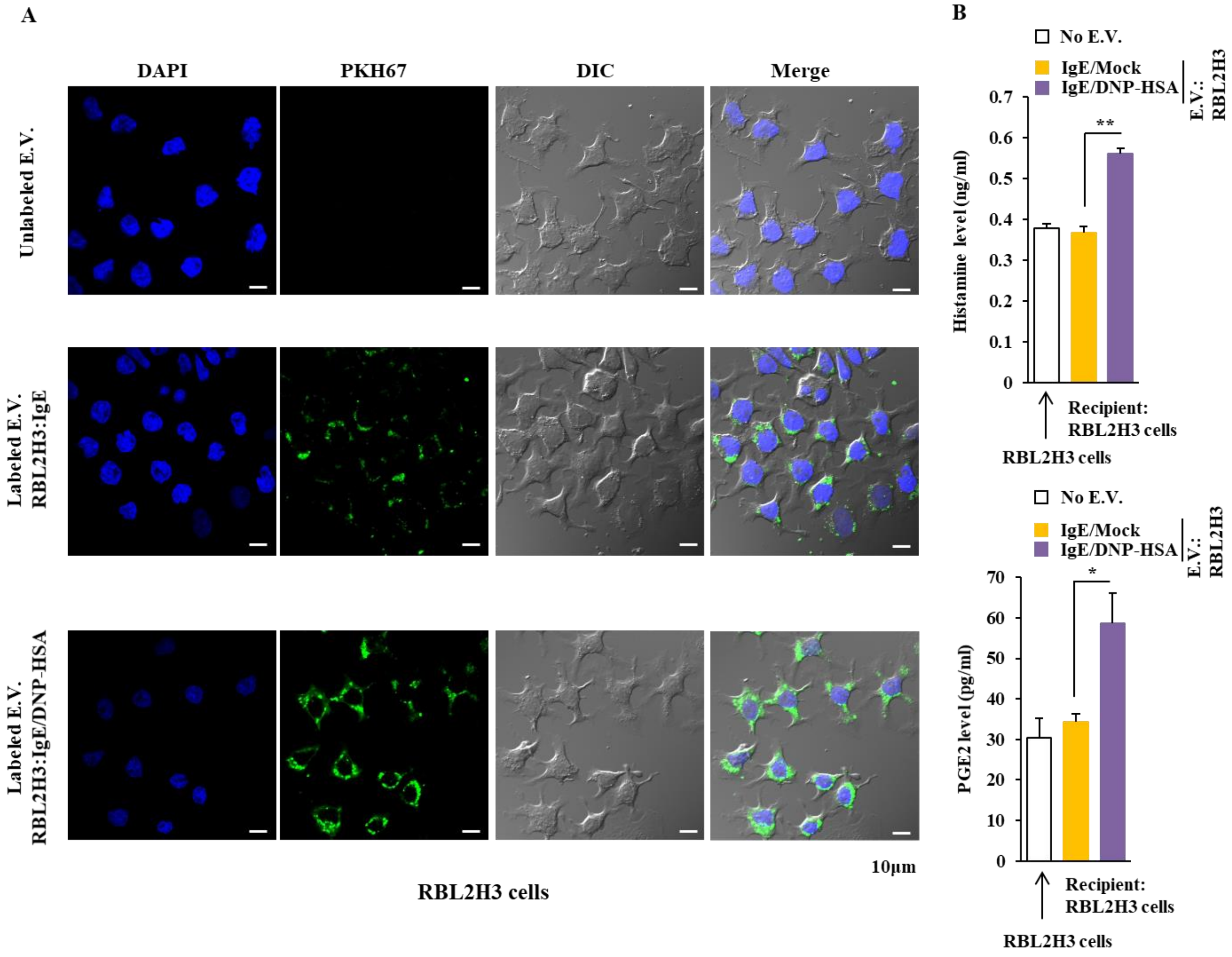

Figure S7. Extracellular vesicles shuttle between cells and induce features of allergic inflammation. (A) Extracellular vesicles isolated from antigen-stimulated RBL2H3 cells were labeled with PKH67 dye (green fluorescence). PKH67-labeled extracellular vesicles were added to RBL2H3 cells. Cells were then visualized using a confocal laser scanning microscope (LSM710). E.V. denotes extracellular vesicles. (B) Extracellular vesicles isolated from RBL2H3 cells unstimulated or stimulated with DNP-HSA for 1 h were added to RBL2H3 cells and incubated for 24 h. Levels of histamine and PGE2 were determined using culture medium. No extracellular vesicles denote levels of histamine released or PGE2 in RBL2H3 cells without extracellular vesicles treatment.

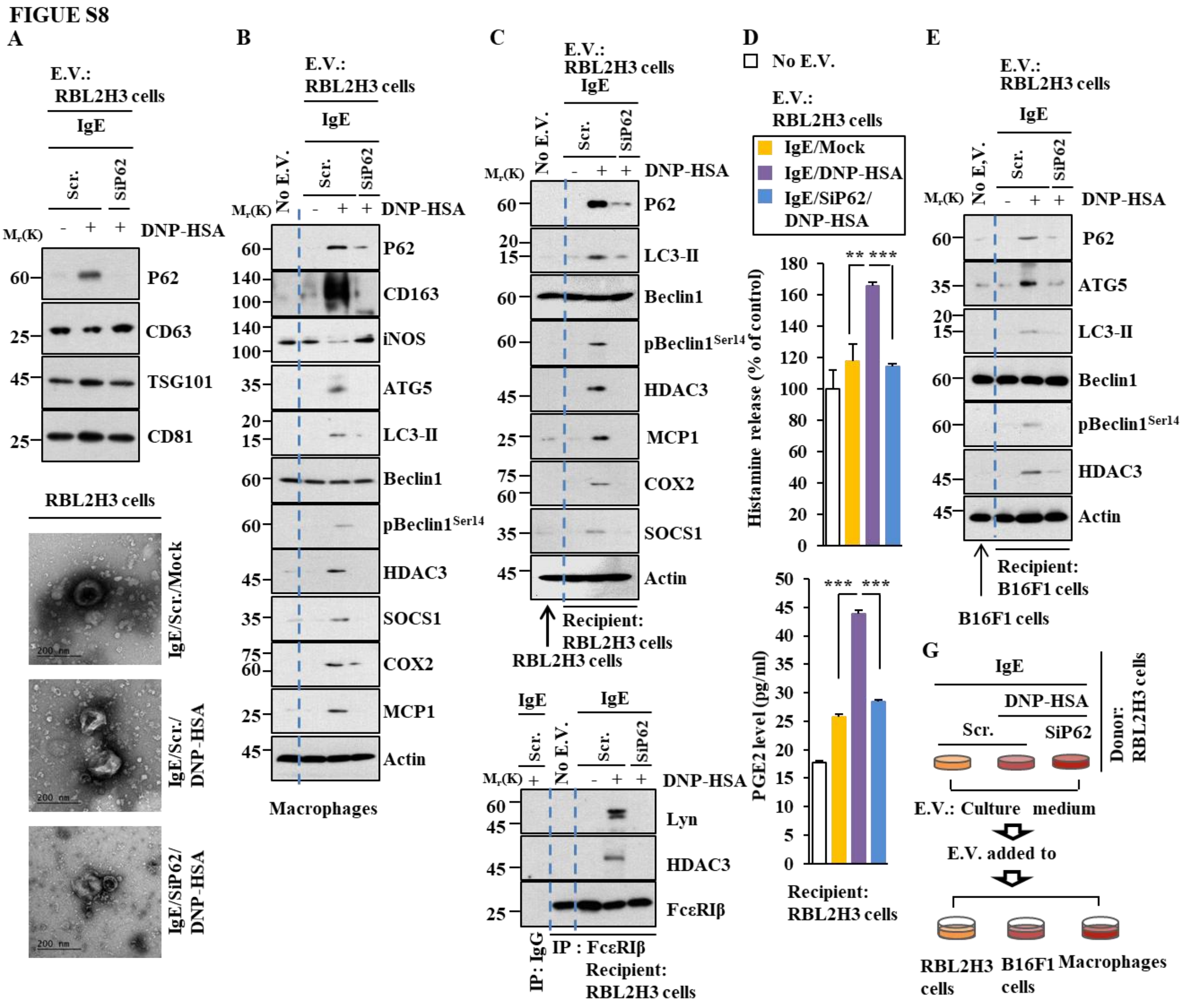

Figure S8. P62 mediates effects of extracellular vesicles on cellular interactions. (A) RBL2H3 cells were transfected with indicated siRNA. The next day, cells were sensitized with IgE for 24 h followed by stimulation without or with DNP-HSA for 1 h. Extracellular vesicles isolated from antigen-stimulated RBL2H3 cells transfected with indicated siRNA were subjected to immunoblot (upper panel). Extracellular vesicles were observed by TEM (lower panel). (B, C) Extracellular vesicles were added to lung macrophages or RBL2H3 cells and incubated for 24 h followed by immunoblot and immunoprecipitation. No extracellular vesicles denote immunoblot of lung macrophages or immunoblot or immunoprecipitation of RBL2H3 cells without extracellular vesicles treatment. (D) Culture medium of RBL2H3 cells was employed for determination of levels of histamine released and PGE2. (E) Extracellular vesicles were added to B16F1cells and incubated for 24 h followed by immunoblot. (F) Same as (E) except that migration and invasion assays were performed. (G) Experimental schemes to examine role of p62 in cellular interactions mediated by exosomes.

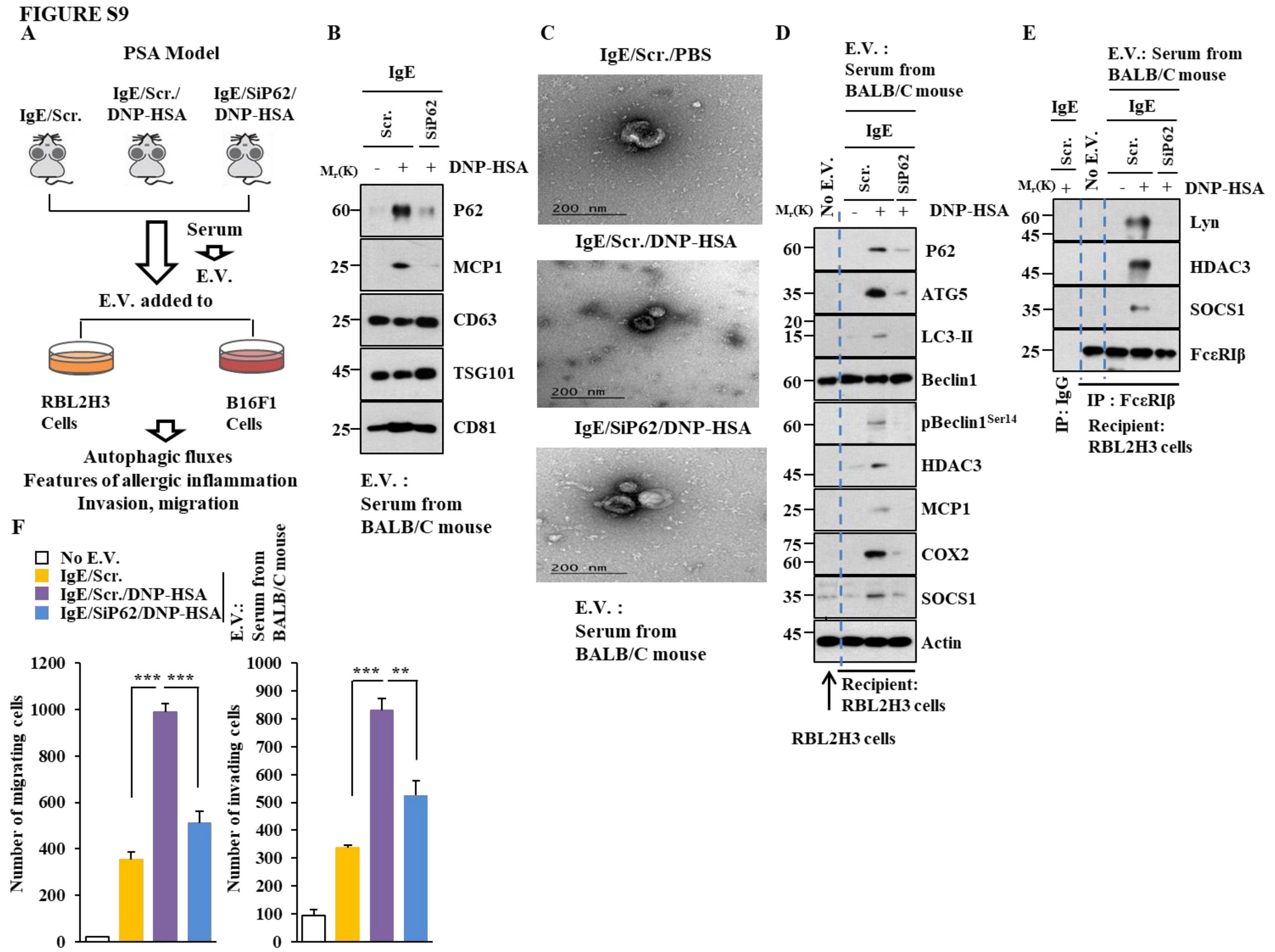

Figure S9. P62 mediates PSA-promoted cellular interactions. (A) Experimental schemes to examine role of p62 in PSA-promoted cellular interactions. Induction of passive systemic anaphylaxis was performed as described. Each mouse received intravenous injection of Scr. or p62 SiRNA at the time of IgE injection. Each experimental group consisted of four BALB/C mice. (B, C) Extracellular vesicles isolated from serum of each mouse of experimental group were subjected to immunoblot and were observed by TEM. (D, E) Extracellular vesicles were added to RBL2H3 cells and incubated for 24 h followed by immunoblot and immunoprecipitation. No extracellular vesicles denote immunoblot or immunoprecipitation of RBL2H3 cells without extracellular vesicles treatment. (F) Same as (D) except that migration and invasion assays were performed.
